# Nanoparticle-Catalyzed Transamination under Tumor Microenvironment Conditions: A Novel Tool to Disrupt the Pool of Amino Acids and GSSG in Cancer Cells

**DOI:** 10.1021/acs.nanolett.3c04947

**Published:** 2024-03-15

**Authors:** Javier Bonet-Aleta, Juan Vicente Alegre-Requena, Javier Martin-Martin, Miguel Encinas-Gimenez, Ana Martín-Pardillos, Pilar Martin-Duque, Jose L. Hueso, Jesus Santamaria

**Affiliations:** 1 Instituto de Nanociencia y Materiales de Aragon (INMA) CSIC-Universidad de Zaragoza, Campus Rio Ebro, Edificio I+D, C/Poeta Mariano Esquillor, s/n, 50018 Zaragoza, Spain; 2 Department of Chemical and Environmental Engineering, University of Zaragoza, Campus Rio Ebro, C/María de Luna, 3, 50018 Zaragoza, Spain; 3 Networking Res. Center in Biomaterials, Bioengineering and Nanomedicine (CIBER-BBN), Instituto de Salud Carlos III, 28029 Madrid, Spain; 4 Instituto de Investigación Sanitaria (IIS) de Aragón, Avenida San Juan Bosco, 13, 50009 Zaragoza, Spain; 5 Departamento de Química Inorgánica, Instituto de Síntesis Química y Catálisis Homogénea (ISQCH), CSIC-Universidad de Zaragoza, C/Pedro Cerbuna 12, 50009 Zaragoza, Spain; 6 Department of Organic Chemistry, University of Zaragoza, 50009 Zaragoza Spain; 7 Surgery Department, Medicine Medical School, University of Zaragoza, 50009 Zaragoza, Spain

**Keywords:** Copper, Amino acids, Transamination, Nanocatalysis, Cancer therapy, Glutathione, Glutamine, Alanine, Pyruvate

## Abstract

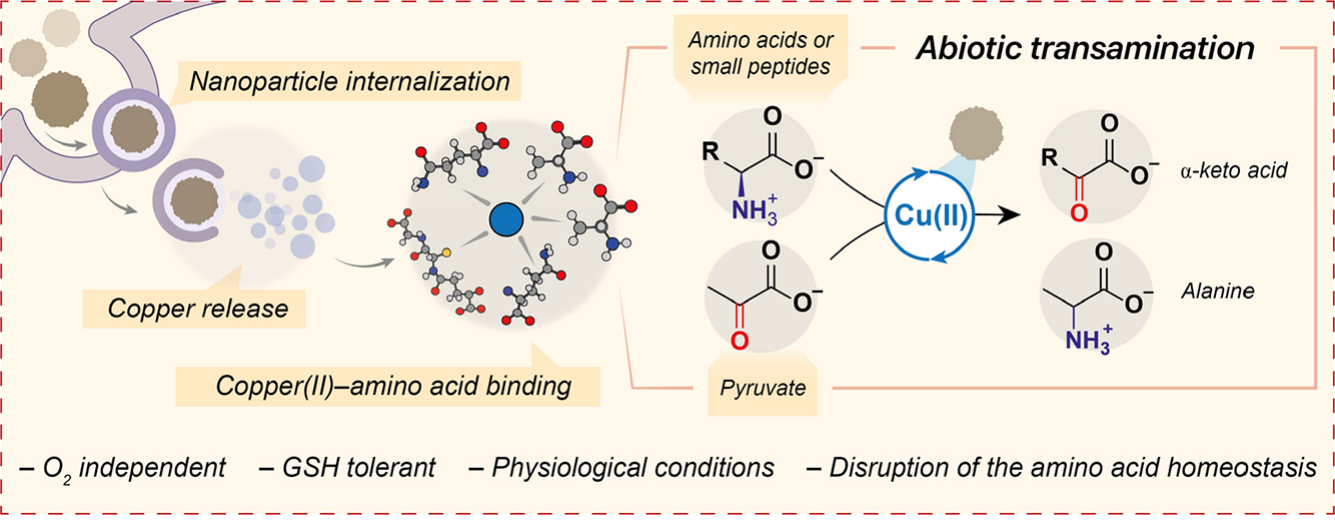

Catalytic cancer therapy targets cancer cells by exploiting the specific characteristics of the tumor microenvironment (TME). TME-based catalytic strategies rely on the use of molecules already present in the TME. Amino groups seem to be a suitable target, given the abundance of proteins and peptides in biological environments. Here we show that catalytic CuFe_2_O_4_ nanoparticles are able to foster transaminations with different amino acids and pyruvate, another key molecule present in the TME. We observed a significant *in cellulo* decrease in glutamine and alanine levels up to 48 h after treatment. In addition, we found that di- and tripeptides also undergo catalytic transamination, thereby extending the range of the effects to other molecules such as glutathione disulfide (GSSG). Mechanistic calculations for GSSG transamination revealed the formation of an imine between the oxo group of pyruvate and the free −NH_2_ group of GSSG. Our results highlight transamination as alternative to the existing toolbox of catalytic therapies.

Catalytic nanoparticles have recently been enlisted as new weapons in the fight against cancer. In particular, it is expected that they will help to modify the chemistry of the tumor microenvironment (TME), fostering tumor cell death or at least a nonproliferative scenario. Catalytic actions followed two main strategies. The first corresponds to the so-called “pro-drug activation” approach that involves *in situ* production of chemotherapy drugs from less toxic or inert molecules, usually by metal-catalyzed chemical reactions^1^ such as dealkylation,^2^ azide–alkyne cycloaddition,^3^ or carbamate cleavage^4^ (Figure 1a). This has given rise to a vast array of possibilities, especially with deprotection chemistry, as researchers devised creative ways of anchoring inactive functional groups that could later be cleaved on site by the action of a metal catalyst.^1,5^ The second strategy, often termed “nanocatalytic therapy”, exploits essential features of the tumor microenvironment (TME)^6−8^ and, in contrast to the pro-drug activation route, rather than introducing foreign molecules, attempts to do chemistry with the chemical species already available. The set of reactions targeted for catalytic therapy has not changed in years, including only four processes, namely, (i) glutathione (GSH) oxidation;^9^ (ii) reactive oxygen species (ROS) production (H_2_O_2_, ^•^O_2_– or ^•^OH);^10,11^ (iii) O_2_ production using endogenous H_2_O_2_,^12^ and (iv) glucose oxidation^13^ (Figure 1b). These reactions interfere with cellular metabolism in general but are particularly harmful to cancer cells given their elevated oxidative stress^14^ and dependency on glucose uptake.^15^*In vitro* and *in vivo* studies have analyzed potential effects on proteins, enzymes, and genes involved in redox homeostasis whose functioning becomes altered by these catalytic processes, such as, glutathione peroxidase (GPX4),^16^ dihydroorotate dehydrogenase (DHODH),^17^ or hypoxia-induced factor (HIF-1)^18^ among others. These catalytic reactions are powerful tools to disrupt tumor homeostasis by altering its redox balance (via processes i and ii), the typically hypoxic environment (via process iii), and nutrient supply (via process iv), respectively. However, reactions i, ii, and iv are oxygen-dependent and are therefore hampered under the hypoxic conditions of the TME. This is where reaction iii enters, aimed to partially alleviate hypoxia locally using available H_2_O_2_, but on many occasions, the reaction rates or the insufficient availability of H_2_O_2_ limit the feasibility of the process. Therefore, unveiling new O_2_-independent processes of therapeutic interest is of paramount interest.

**Figure 1:**
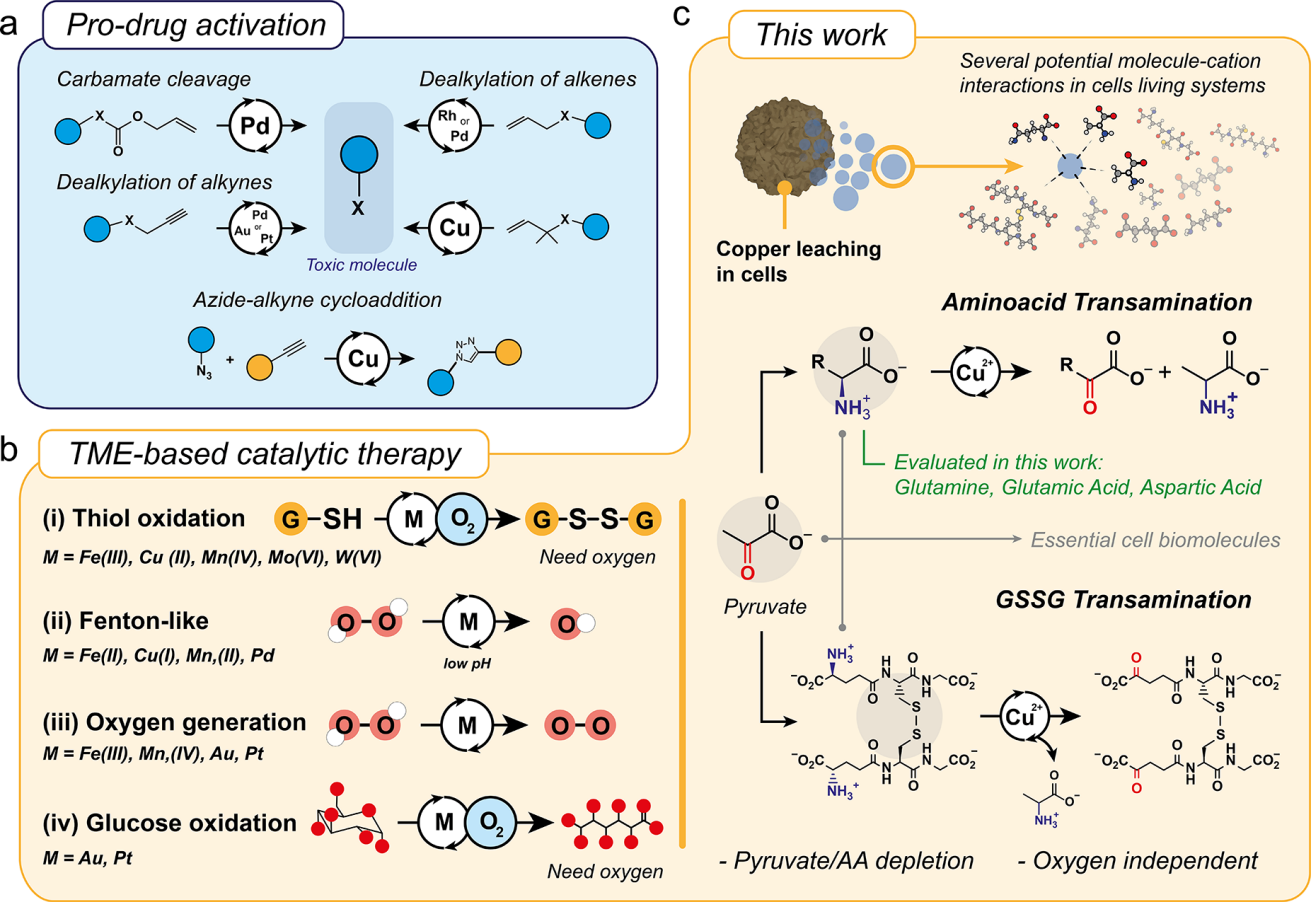
Different nanoparticle-catalyzed strategies developed for cancer therapy. (a) Bioorthogonal catalysis based on pro-drug activation strategies typically require a transition-metal catalyst including Pd, Pt, Au, Rh, or Cu to form a cytotoxic compound by either removing a chemical group from a pro-drug or binding two low-toxicity molecules. (b) Reactions employed in the context of nanocatalytic therapy in the TME: (i) GSH oxidation, (ii) ^•^OH or (iii) O_2_ generation using endogenous H_2_O_2_, and (iv) glucose oxidation. (c) New scenario potentially enabled by the internalization of transition-metal leaching nanoparticles. In particular, Cu^2+^ catalyzes the transamination reaction between the −NH_3_^+^ group attached to α-C of an amino acid/peptide and the keto group of pyruvate to yield d/l-alanine and the corresponding keto-acid derived from the amino acid/peptide. Reactions target key biomolecules in the cell: glutamine, glutamic acid, aspartic acid, and GSSG.

As already mentioned, the existing nanoparticle catalysts for catalytic therapy work exclusively around the four processes described in Figure 1b, and no new reaction pathways have been reported. This is surprising since the TME is teeming with key molecules and reactions that could be exploited, and it seems unlikely that these four processes could exhaust all of the catalytic opportunities available to fight cancer growth. Recently, Moran and co-workers^19,20^ demonstrated that transamination, a reaction of biological importance that is usually governed by enzymatic catalysis, could also be catalyzed in a test tube by Co^2+^, Ni^2+^, V^5+^, and especially Cu^2+^ cations. Inspired by this work, we hypothesized that the use of Cu-releasing nanoparticles that are easily internalized by endocytosis and display a rapid Cu^2+^ release kinetics^21^ could effectively perform this type of catalysis within cancer cells, thus adding a new reaction to the panoply pictured in Figure 1b. Specifically, we propose that the catalytic action of transition metals on key molecules such as amino acids and α-ketoacids (Figure 1c) may open up unexplored therapeutic opportunities.

Especially noteworthy, in view of its potential therapeutic value, is the oxygen-independent character of the transamination processes, enabling them to occur within the hypoxic TME without the need to resort to complex oxygen-generation schemes. Cancer cells are particularly sensitive to the depletion of key molecules employed as building blocks to sustain the energetic and growth demands of their accelerated metabolism.^22^ Amino acids are essential players in these metabolic routes, and indeed, amino acid starvation is currently employed in the clinic to treat acute lymphoblastic leukemia or non-Hodgkin lymphoma by targeting asparagine through the enzyme l-asparaginase,^23^ while others like glutamine, arginine, or methionine are currently being explored in preclinical or clinical phases.^24^ Finally, as presented in Figure 1b, glutathione (GSH) is rapidly becoming another therapeutic target due to its central role in balancing intracellular redox stress in cancer cells. Transition-metal catalysis^9^ converts GSH into glutathione disulfide (GSSG).^21,25^ Unfortunately, this process can be easily reverted through the action of glutathione reductase,^26^ thereby reducing the therapeutic effect of the oxidation. In contrast, the transamination process of Figure 1c could provide another, nonoxidative way to deplete both GSH and GSSG pools since they have an iso-peptidic bond between the −COO^–^ group from the side chain of the glutamate residue and the amino group from cysteine that is expected to be chemically able to undergo transamination.

Using CuCl_2_ as the Cu(II) source requires a previous reduction step into Cu(I)^27^ to be internalized through the high-affinity Cu(I)-selective copper ion channel (CTR1),^28^ which restricts the total copper uptake by cell. Instead, here, we have used CuFe_2_O_4_ nanoparticles as reservoirs to deliver much larger amounts of Cu upon internalization into U251-MG glioblastoma cells. Nanoparticle internalization typically occurs via an endocytosis^29−31^ and this enables higher internalization rates. Specific Cu importers such as members of the Ctr1 family have evolved to maintain Cu homeostasis and, therefore, manage very low ion fluxes. In contrast, the primary particle size of the CuFe_2_O_4_ nanoparticles is around 8 nm and the specific Cu intake in each internalization event is likely to be much larger, as in the culture medium the particles form 37 nm agglomerates, according to NTA results.^21^ This means that internalization of Cu-containing nanoparticles is highly efficient to supply Cu to the cell, and in fact, agglomerates of CuFe_2_O_4_ NPs are easily visible in confocal microscopy images after a few hours of incubation.^29^ Herein, we investigate whether the Cu^2+^ cations leached from the NPs, besides promoting GSH oxidation,^21,29^ may also catalyze transamination reactions using pyruvate and different amino acids/peptides as substrates. In addition, we have performed DFT calculations to provide a theoretical support to the catalytic outcomes observed and specifically to the variation of intracellular glutamine, as a key amino acid, together with alanine, as the transamination reaction product. We also demonstrate a promising and nonpreviously reported specific transamination of GSH, thereby adding a new reaction that may target this antioxidant key for the regulation of redox homeostasis in cancer cells.

We first carried out kinetic studies of the removal of glutamine, glutamic acid, and aspartic acid via transamination, although in this case we used CuFe_2_O_4_ nanoparticles as catalysts^19^ (information regarding the synthesis and characterization of CuFe_2_O_4_ nanoparticles can be found in the Supporting Information and Figure S1). The selection of this nanostructure was motivated by two crucial reasons. First, this catalyst exhibits exceptional performance in GSH oxidation due to the synergy between Cu and Fe catalysis.^21^ Second, this specific partnership works particularly well under TME conditions (i.e., hypoxia and low pH^10−12^). Therefore, we hypothesize that CuFe_2_O_4_ nanoparticles can operate in a sequential manner. Initially, the high concentration of GSH in cancerous cells can trigger the release of copper ions from CuFe_2_O_4_. Subsequently, these copper ions can rapidly deplete GSH levels through oxidation catalysis, aided by the presence of the remaining Fe-enriched nanoparticle. Finally, the copper ions might catalyze a transamination reaction using endogenous amino acids/peptides and pyruvate as substrates. Consequently, all tested reactions in our system were carried out in the presence of 5 mM of GSH, an expected intracellular concentration in tumor cells.^29^ This is important because the presence of GSH promotes Cu^2+^ leaching, to a much larger extent than any of the amino acids tested in this work (Figure S2). Additionally, the catalyst showed negligible leaching in the absence of GSH/amino acids (Figure S3).

Regarding the transamination of glutamine, the primary amine of glutamine (highlighted in blue, Figure 2a) undergoes exchange with the keto group of pyruvate (highlighted in red, Figure 2a) yielding the corresponding α-ketoacid derived from the amino acid and alanine (Figure 2a). This reaction is catalyzed by the released Cu^2+^ from the CuFe_2_O_4_ NPs.^21^ It is noteworthy that this reaction is nonstereospecific^19^ and, consequently, the d-alanine generated as byproduct (half of the total produced) becomes useless for cells. Therefore, the transamination reaction depicted in Figure 2a is potentially useful for a catalytic starvation therapy scenario since it simultaneously removes glutamine and pyruvate, two key molecules in different types of cancers,^32,33^ and can perform in an oxygen-independent ambient atmosphere.^34^ Afterward, we monitored the reaction between the CuFe_2_O_4_ NPs and glutamine at different times (Figure 2b–d). ^1^H NMR analysis at early reaction stages (5 h, Figure 2b) revealed the characteristic signal of the generation of GSSG as byproduct of the GSH oxidation.^21,35^ In contrast, we could not detect alanine, suggesting that kinetics of Cu^2+^-catalyzed GSH transformation into GSSG are faster in comparison to the Cu^2+^-catalyzed transamination reaction.

**Figure 2:**
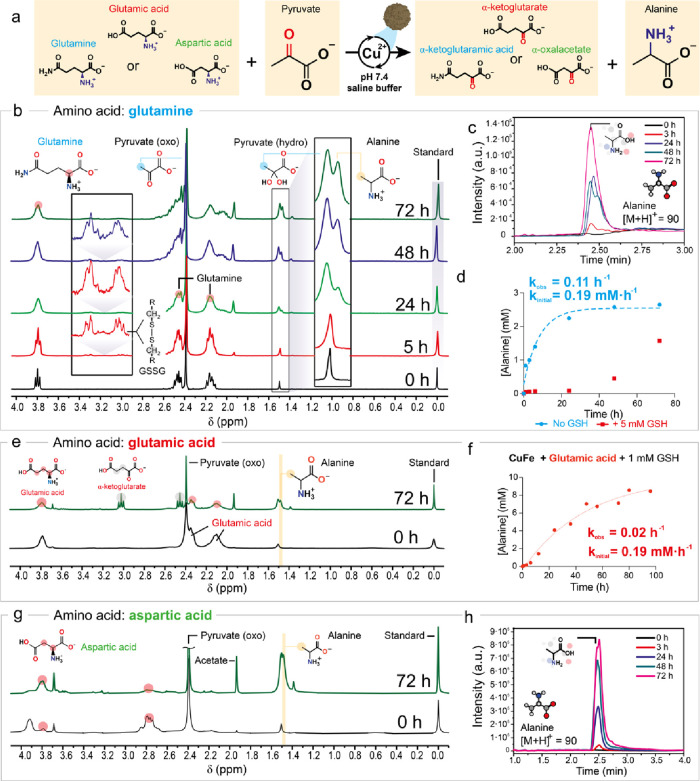
^1^H NMR analysis of the transamination reaction in the presence of CuFe_2_O_4_ nanoparticles: (a) Schematic display of the transamination reaction between selected amino acids (glutamine, glutamic acid, and aspartic acid acting as amino donors) and pyruvate to yield α-ketoacid acid and alanine. (b) ^1^H NMR analysis of the glutamine–pyruvate transamination reaction at different times. (c) UPLC-MS chromatogram of the produced alanine from the glutamine–pyruvate transamination (*m*/*z* = 90, [M + H]^+^). (d) Control experiment using CuFe_2_O_4_ as catalyst in the absence of GSH using glutamine as the amino acid substrate. (e) ^1^H NMR spectra of the glutamic acid–pyruvate transamination at different reaction time intervals. (f) Evolution of alanine concentration with reaction time in a system containing 1 mM GSH using glutamic acid as the amino acid substrate (additional ^1^H NMR spectra and UPLC-MS chromatograms can be found in Figure S5 and Figure S6). Alanine derived from aspartic acid–pyruvate transamination reaction was also found in (g), the ^1^H NMR spectra corresponding to the aspartic acid–pyruvate transamination at different reaction time intervals. (h) UPLC-MS analysis of alanine derived from aspartic acid–pyruvate transamination (additional ^1^H NMR spectra/UPLC-MS chromatograms are depicted in Figure S6 and Figure S7). Reaction conditions for all experiments: [Cu] = 6 mM, [pyruvate] = 30 mM, [amino acid] = 45 mM, [GSH] = 5 mM, pH = 7.4 (Na_2_HPO_4_/NaH_2_PO_4_ 1M), *T* = 37 °C.

More extended reaction times resulted in an increase in the alanine signal (CH_3_, 1.48 ppm, Figure S4), also detected by UPLC-MS (Figure 2c), and α-ketoglutaramic acid (Figure S6), the α-ketoacid derived from glutamine. The reaction also took place in the absence of GSH (Figure 2d, Figure S5), since glutamine itself has the potential to release copper cations (Figure S2), which are the catalytically active species in the transamination reaction.^19^ We also tested transamination with other important single amino acids, such as glutamic acid (Figure 2e,f, Figure S7 and Figure S8) and aspartic acid (Figure 2g,h, and Figure S9 and Figure S10). The results were analogous to glutamine. ^1^H NMR and UPLC-MS analyses revealed a time-dependent increase of alanine and the corresponding α-ketoacid (except for the case of the oxalacetate produced from aspartic acid, which can be easily decarboxylated in the presence of transition metals such as Cu^2+^ ^36^), together with the depletion of pyruvate and the donor amino acid (Figures S7–S10). No alanine was detected in the absence of the CuFe_2_O_4_ NPs (Figure S11).

The reaction rates depended on the amino acid employed as substrate, with initial kinetic constants (*k*_initial_) sorted in the following order: aspartic acid > glutamic acid ≈ glutamine (Figure S12) in agreement with the results of Mayer et al.^19^ using CuCl_2_. The generation of alanine using the CuFe_2_O_4_ NPs was lower than that of the control experiments with CuCl_2_ as catalyst (Figure S13), but the observed kinetic constant (*k*_obs_) exhibited similar values. This can be attributed to the immediate availability of copper when CuCl_2_ is utilized, in contrast to the progressive release taking place in the case of the CuFe_2_O_4_ nanoparticles (Figure S2). We also observed that the concentration of GSH played a key role in the reaction kinetics. Decreasing the initial concentration of GSH from 5 mM (expected intratumoral levels) to 1 mM positively affected the progress of the reaction (Figure 2f), reducing the activation time and increasing the total alanine produced, in comparison to the experiments where the initial concentration of GSH was 5 mM (Figure S12b). Two additional control experiments were conducted to delve deeper into the role of GSH. First, the presence of 1 mM GSH also hampered the progression and yield of transamination using CuCl_2_ as catalyst (Figure S14). Second, the absence of GSH enhanced the overall yield and kinetics of the transamination reaction of glutamine using CuFe_2_O_4_ (Figure 2d). This system was chosen because glutamine itself can also release copper from CuFe_2_O_4_ (Figure S2), although to a minor extent in comparison to GSH. Besides GSH, we also tested the influence of another biologically relevant sulfhydryl containing molecule, cysteine, known by its affinity toward copper ions.^37^ In the presence of 100 μM of cysteine, a concentration equal to intracellular levels,^38^ the transamination reaction could also occur, producing alanine since early reaction times (Figure S15).

A higher concentration of GSH increases the reducing character of the mixture, decreasing the availability of Cu^2+^ ions in solution,^21^ which is the active species in the transamination reaction.^19^ In contrast, if the amino acid employed as substrate does not induce copper release, a small amount of GSH or of another suitable agent is required to trigger this release. Finally, the transamination reaction using CuO nanoparticles rendered similar *k*_initial_ values to CuFe_2_O_4_, reinforcing the general applicability of any copper-containing nanostructure able to release Cu(II) cations for this reaction (Figure S16).

We also hypothesized that the same transamination could extend beyond single amino acids to include peptides that possess free carboxyl (−CO_2_^–^) and amino (−NH_3_^+^) groups linked to the α-C atom of an amino acid residue. A very important example of this family of compounds would be GSH and its oxidized form GSSG. Both are peptides that exhibit this specific structural configuration (highlighted in orange in Figure 3a). Given their central role in redox homeostasis in cancer cells, transamination of these molecules could have significant interest in cancer therapy because the resulting unnatural α-ketoGSSG product might be more challenging for cancer cells to metabolize compared to naturally occurring antioxidants like GSH and GSSG.^39^

**Figure 3:**
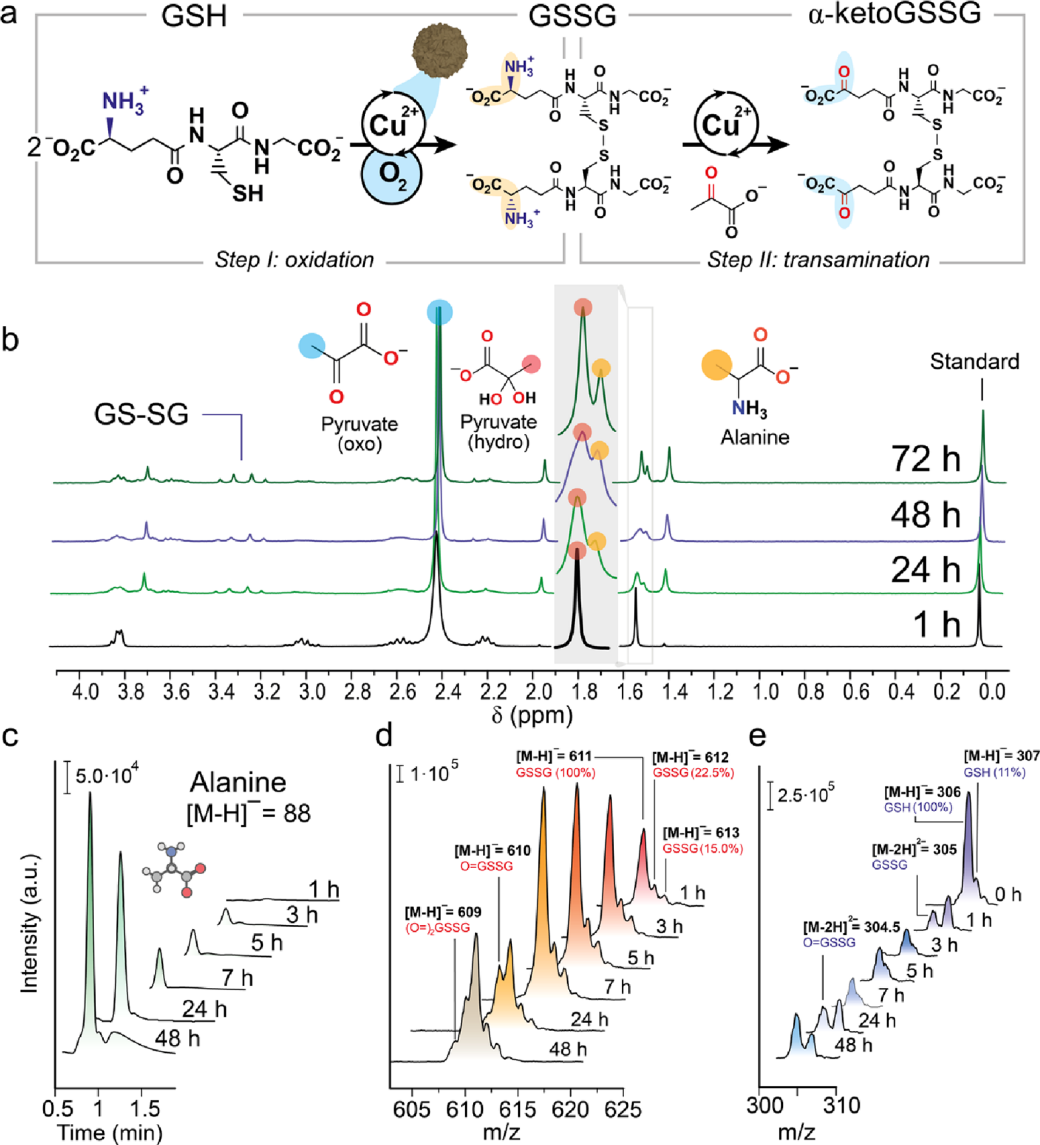
Cu-catalyzed transamination of GSH-GSSG in the presence of CuFe_2_O_4_ nanoparticles. (a) Cu^2+^ released from CuFe_2_O_4_ nanoparticles first catalyzes GSH oxidation with dissolved O_2_, giving GSSG; then it furthers catalyzes its transamination with pyruvate. (b) ^1^H NMR and (c) UPLC-MS analysis of the generation of alanine from transamination of GSSG at different reaction times. (d, e) MS analysis of the formation of α-ketoGSSG and the depletion of GSH at various reaction times. Reaction conditions: [Cu] = 6 mM, [pyruvate] = 30 mM, [GSH] = 5 mM, pH = 7.4 (Na_2_HPO_4_/NaH_2_PO_4_ 1 M), *T* = 37 °C.

In the presence of O_2_, the Cu^2+^-catalyzed oxidation of GSH to GSSG exhibited faster kinetics than the competing transamination, as can be seen at early reaction times (1 h), where UPLC-MS analysis revealed predominant generation of GSSG (Figure 3d) and depletion of GSH (Figure 3e), with minimal formation of alanine through transamination (Figures 3b,c). However, at longer reaction times (24 h and beyond), the UPLC-MS, ^1^H NMR, and MS analyses detected the formation of alanine and α-ketoGSSG, demonstrating successful transamination of GSSG (Figures 3b–d). Additional experiments confirmed the compatibility of other polypeptides, such as γ-Glu-ε-Lys with this transformation (Figure S17). Moreover, it was experimentally determined that amino acids containing secondary amines, such as proline, were not transaminated (Figure S18).

The transamination mechanism was investigated by using density functional theory (DFT). Due to the presence of strong interactions among the different components, a direct evaluation of the Gibbs free energy difference (Δ*G*) between the isolated reagents and products did not yield informative results (Figure S19). Consequently, we used a comprehensive modeling approach that encompassed all pertinent reaction components, including GSSG/α-ketoGSSG, Cu^2+^, pyruvate/alanine, and HPO_4_^2–^, in the calculations illustrated in Figure 4.

**Figure 4:**
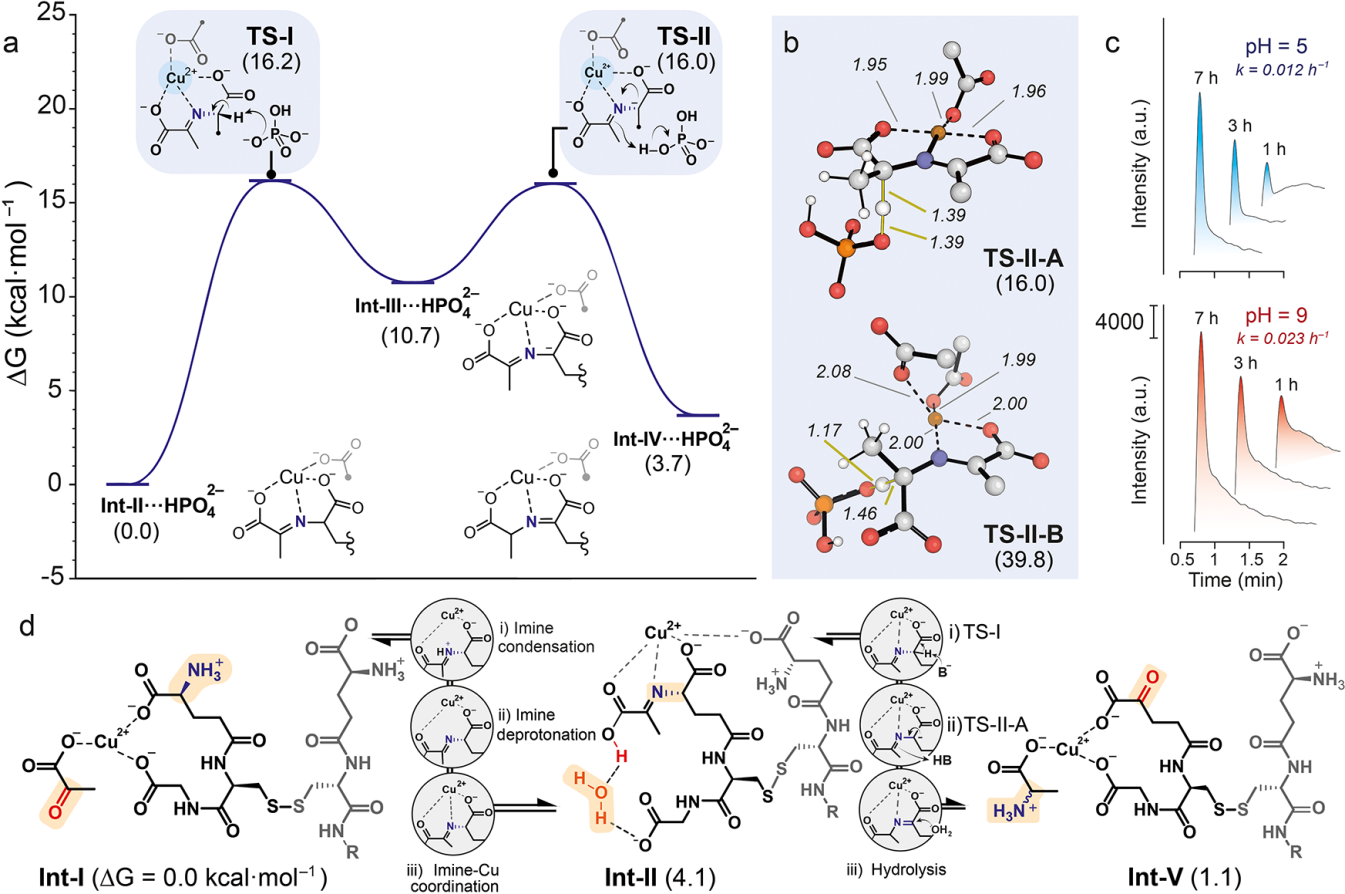
(a) Δ*G* values for the 1,3-H shift with HPO_4_^2–^ acting as the H-transferring agent. (b) Depiction of the most stable conformers of **TS-II A** and **TS-II B**. Dotted black lines indicate Cu–ligand bonds, thin yellow lines represent TS bonds, and distances are displayed in Å. (c) Experimental reaction rates at different pH values after 7 h. (d) Thermodynamic aspects of the transamination process. Computational protocols: DFT calculations^40−42^ were carried out with ωB97X-D/Def2-QZVPP//ωB97X-D/6-31+G(d,p),^43−51^ SMD^52−57^ (solvent = water) was included in all the calculations, standard state = 1 M, *T* = 37 °C.^58^

Initially, Cu^2+^ establishes strong interactions with the −CO_2_^–^ groups present in GSSG and pyruvate (**Int-I**, Figure 4d). In the most stable conformation discovered, the cation predominantly coordinates with three −CO_2_^–^ groups, two of which stem from a folded branch of GSSG, and the other from pyruvate. Subsequently, one of the −NH_3_^+^ groups of GSSG reacts with the ketone of pyruvate, resulting in the formation of a protonated imine that requires deprotonation before coordinating with Cu to generate **Int-II**. This sequence of steps exhibits slight endergonicity (Δ*G* = 4.1 kcal·mol^–1^) when one of the −CO_2_^–^ groups acts as the base for deprotonation to obviate the need for separate calculations. Consistent with previous Cu-catalyzed transaminations,^19^ the calculated rate-limiting step involves the presence of the activated imine···Cu group. Therefore, the pH employed could influence the reaction kinetics as a more alkaline solution would render deprotonation and subsequent imine···Cu coordination. This theoretical approach is supported by the observed 2-fold reaction rate increase upon raising the pH from 5 to 9 (Figure 4c). Furthermore, the computational findings indicate that the transamination equilibrium is essentially energetically balanced (Δ*G* = 1.1 kcal·mol^–1^, Figure 4d), emphasizing the importance of an excess of pyruvate in the medium to drive the equilibrium toward the α-ketoGSSG product.

Similar to previous mechanisms calculated for metal-catalyzed transaminations,^19^ the kinetics of the process are governed by a stepwise 1,3-H migration. In this mechanism, an HPO_4_^2–^ basic molecule from the solution buffer triggers an H shift from the α-C atom to the iminic C atom (Figure 4a). As in the imine-Cu coordination process, this base-promoted 1,3-H migration should be favored by more basic pH environments, which aligns with the experimental kinetic trend (Figure 4c). Interestingly, this process involves not only C=N···Cu activation but also CO_2_^–^···Cu activation of the −CO_2_^–^ group located next to the carbanion resulting from H abstraction. The Cu atom coordinates with this −CO_2_^–^ group, enhancing its capability to stabilize neighboring carbanions and thus reducing the energy barriers (Δ*G*^‡^ from 39.8 to 16.0 kcal in **TS-II B** and **TS-II A**, respectively, Figure 4b).

Encouraged by the activity of lixiviated Cu^2+^ as a transamination catalyst for a variety of biologically relevant substrates, we evaluated the potential of CuFe_2_O_4_ NPs to disrupt the amino acid pool in U251-MG cancer cells. We selected these cells because of the significant role of glutamine in their metabolism.^59,60^ Indeed, some studies point out the relevance of glutamine in NADPH production and anaplerotic reactions (i.e., to generate Kreb’s cycle intermediates) beyond their role as a nitrogen source in glioblastoma cells.^61^ We detected a significant decrease in the intracellular glutamine levels in U251-MG cells incubated with CuFe_2_O_4_ NPs after 72 h (Figure 5a). On the other hand, intracellular alanine levels clearly increased for the CuFe_2_O_4_-treated group up to 48 h. This is consistent with the results shown above, as there is a significant pool of intracellular pyruvate and using pyruvate as the ketoacid always yielded alanine in all transamination reactions evaluated throughout this work, regardless of the amino precursor. Interestingly, alanine was consumed in both the control and treated U251-MG cells (Figure 5b) at 72 h. We attribute this result to cellular metabolic responses. In the absence of other amino acids, alanine can be incorporated into the TCA cycle to support ATP biosynthesis through its enzymatic transamination to pyruvate.^62^ In addition, alanine can also serve as nitrogen source in glutamine-starved glioblastoma cells.^63^ Both of these facts may explain the strong decrease observed in both groups after 72 h. Finally, although only glutamine was monitored, our results show that the catalyst promotes transamination of a variety of amino acids and peptides, causing a severe disruption of the cell metabolism.

**Figure 5:**
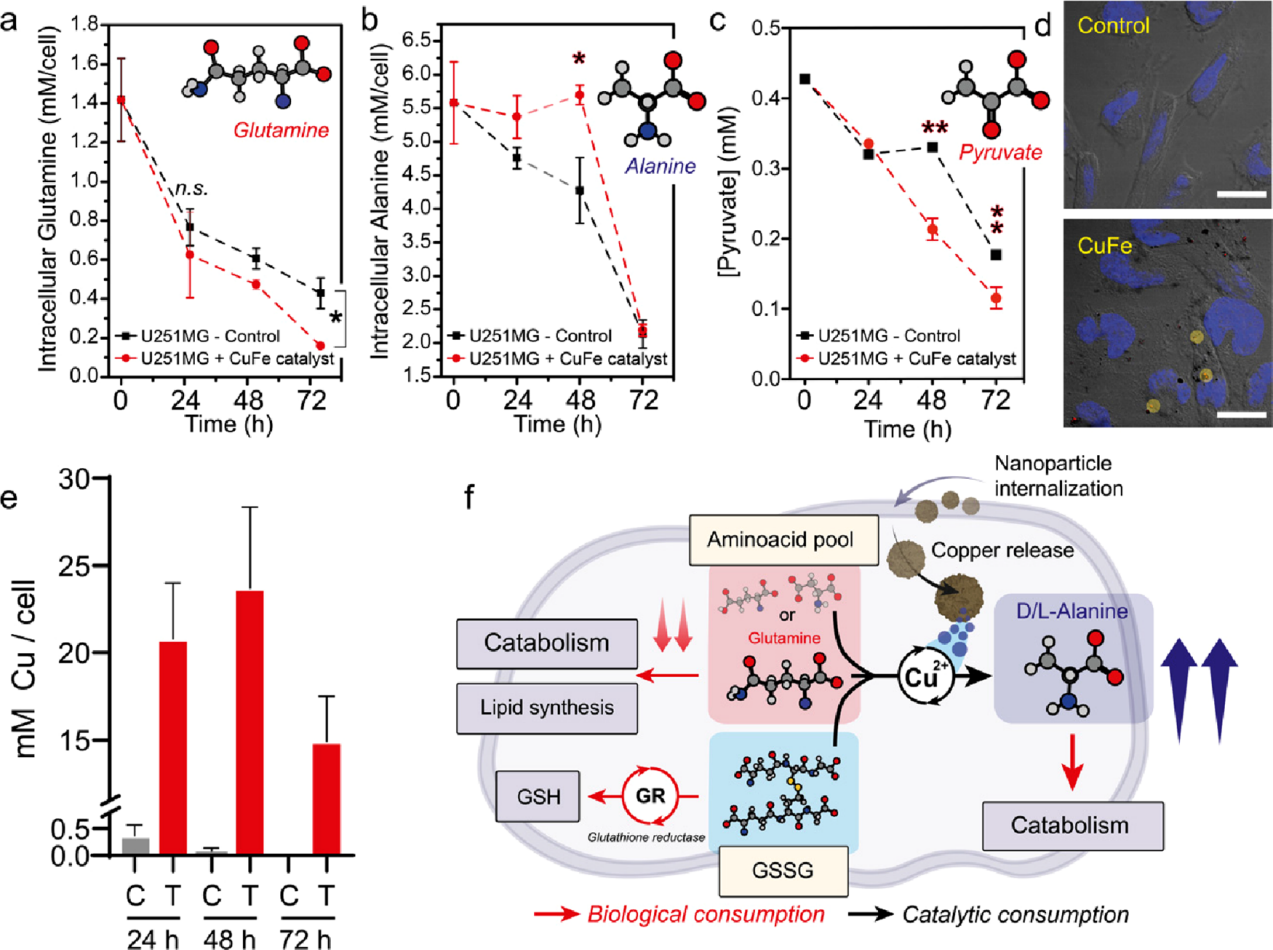
Tracking the intracellular transamination induced by CuFe_2_O_4_ nanoparticles. (a) Intracellular glutamine concentrations decrease for both control and treated U251-MG cells. Glutamine is a key metabolite for cells as one of the major nitrogen sources and is used both for the TCA cycle or for fatty acid/nucleotide biosynthesis.^63^ Treatment with CuFe_2_O_4_ nanoparticles significantly decreased glutamine levels especially after 72 h. (b) Monitoring intracellular alanine concentration revealed different profiles in control/treated U251-MG cells. After 24 and 48 h, the alanine concentration was significantly larger for treated U251, suggesting that artificial transamination had been successfully induced. (c) Pyruvate concentration present in cell media at different incubation times with 0.05 mg·mL^–1^ CuFe_2_O_4_ nanoparticles. As a result of the Cu-catalyzed transamination reaction, the concentration of pyruvate was lower after 48 and 72 h in comparison to the control experiment. Intracellular pyruvate could not be determined with sufficient accuracy due to low concentration levels. (d) Confocal microscopy analysis of U251-MG cells revealed the internalization of CuFe_2_O_4_ nanoparticles in the form of aggregates (highlighted in yellow). (e) Intracellular copper levels of U251-MG cells treated with 50 μg·mL^–1^ of CuFe_2_O_4_ showed a strong increase of copper concentration up to 48 h, followed by a decrease at 72 h. (f) Schematic illustration of some possible catalytic pathways of the intracellular amino acid pool: glutamine (or other amino acids) can enter different metabolic routes to enable ATP or lipid biosynthesis. However, internalization of CuFe_2_O_4_ nanoparticles increases the intracellular concentration of Cu^2+^, a catalyst that promotes artificial amino acid/pyruvate transamination, as well as that of other species with suitable chemical structure such as GSH and GSSG. For GSSG this reaction competes with the reduction of GSSG to GSH by glutathione reductase. Statistically significant differences were expressed as follows: *p < 0.05, **p < 0.005, ***p < 0.0005, and ****p < 0.00005.

We also monitored the concentration of pyruvate, the α-keto acid that acts as substrate for the Cu-catalyzed transamination reaction, in cell media (Figure 5c). We found a larger consumption of pyruvate in the presence of 0.05 mg·mL^–1^ CuFe_2_O_4_ catalyst, which reinforces the idea of a transamination reaction in the biological environment. The intracellular GSH levels in the presence of the CuFe_2_O_4_ NPs dropped after 24 and 48 h in comparison to those in control experiments (Figure S20a). Likewise, the GSSG signal, the main product of GSH oxidation, in cell media progressively increased after nanoparticle treatment (Figure S20b). All these changes in the concentration of glutamine, alanine, pyruvate, and GSH help to explain the abrupt interruption seen in the growth of cells for the treatment (Figure S21) after 24 h. We evaluated the cell viability using nanoparticles prepared by an identical procedure but without containing copper (i.e., iron oxide). As can be seen in Figure S22, a significant decrease of viability is detected for Cu-containing nanoparticles at every concentration studied. The successful internalization of the CuFe_2_O_4_ nanocatalyst was confirmed by confocal microscopy (Figure 5d). Nanoparticle aggregates could be detected inside U251-MG cells due to their own reflection close to the cellular nucleus stained in blue (Figure 5d). We also studied the evolution of total intracellular Cu, the main homogeneous catalysts studied in this work, after treatment with CuFe_2_O_4_ NOs using MW plasma atomic emission spectroscopy (MP-AES) (Figure 5e). The maximum intracellular copper value was reached at 48 h after the treatment with 50 μg·mL^–1^ CuFe_2_O_4_, which is in agreement with the maximum concentration of intracellular alanine in treated U251-MG cells (Figure 5b). After 72 h, the intracellular Cu concentration decreased down to 14.5 mM, following cellular regulation mechanisms and excretion of remaining nanoparticles via endosomes or copper ions via protein exporters such as ATP7A/B.^27^ However, 72 h gives ample time for Cu^2+^ to perform catalysis using the amino acid pool or the cytosolic GSSG as amino donors, and the pyruvate as α-ketoacid to catalyze the transamination reaction (Figure 5f).

In summary, Cu^2+^ cations released from CuFe_2_O_4_ nanoparticles can catalyze transamination using glutamine, glutamic acid, and aspartic acid as amino acid substrates under conditions relevant to TME (i.e., hypoxia and a 5 mM concentration of GSH). The scope of transamination extends to tri- and dipeptides as suitable substrates if an α-C with a free −COO^–^/–NH_3_^+^ group is present in their structure. We have shown experimentally that GSSG can also be subjected to transamination as a result of the formation of an imine between the oxo group of pyruvate and the free −NH_2_ group of GSSG, followed by the coordination of the imine to Cu(II). Lastly, we have observed that glutamine consumption is accelerated while intracellular alanine levels rise and cell proliferation abruptly stops, a scenario in good agreement with transamination reactions catalyzed by Cu(II)-releasing nanoparticles. In summary, the results of this work establish copper-catalyzed transamination as a new valuable reaction to be added to the existing toolkit of TME-directed nanocatalytic therapy.

## Supporting Information Available

Additional information about nanoparticle synthesis, copper leaching tests, transamination experiments with glutamine, glutamic acid, aspartic acid, proline, glutathione disulfide and γ-Glu-ε-Lys, DFT calculations, evolution of GSH and GSSG in biological media and toxicity of control iron nanoparticles; figures of TEM images, size distribution, XRD pattern, evolution of copper vs time, copper release kinetics, ^1^H NMR spectra, UPLC-MS chromatograms, production of alanine at different times, Gibbs free energies, evolution of intracellular GSH, Variation of GSSG signal, cell number vs incubation time, cell viability; AQME data (PDF)
