# Supplementary material for: Nanoparticle-Catalyzed Transamination under Tumor Microenvironment Conditions: A Novel Tool to Disrupt the Pool of Amino Acids and GSSG in Cancer Cells
Source: Nano Lett. 2024 Mar 15;24(14):4091–100. doi: 10.1021/acs.nanolett.3c04947 (PMC11010231; doi:10.1021/acs.nanolett.3c04947)
Supplement: Supplementary file 1 — nl3c04947_si_001.pdf [file nl3c04947_si_001.pdf]

# Supporting Information for

# Nanoparticle-catalyzed transamination under tumor microenvironment conditions: a novel tool to disrupt the pool of amino acids and GSSG in cancer cells

*Javier Bonet-Aleta[1,2,3,4], Juan Vicente Alegre-Requena[5], Javier Martin-Martin[1,6], Miguel Encinas-Giménez[1,2,3,4], Ana Martín-Pardillos[1,2,4], Pilar Martin-Duque[3,4,7], Jose L. Hueso[1,2,3,4,*], Jesus Santamaria[1,2,3,4,*]*

[1]Instituto de Nanociencia y Materiales de Aragon (INMA) CSIC-Universidad de Zaragoza, Campus Rio Ebro, Edificio I+D, C/ Poeta Mariano Esquillor, s/n, 50018, Zaragoza, (Spain).

[2]Department of Chemical and Environmental Engineering, University of Zaragoza, Campus Rio Ebro, C/María de Luna, 3, 50018 Zaragoza (Spain).

[3]Networking Res. Center in Biomaterials, Bioengineering and Nanomedicine (CIBER-BBN), Instituto de Salud Carlos III; 28029 Madrid (Spain)

[4] Instituto de Investigación Sanitaria (IIS) de Aragón, Avenida San Juan Bosco, 13, 50009 Zaragoza, Spain.

[5]Departamento de Química Inorgánica, Instituto de Síntesis Química y Catálisis Homogénea (ISQCH) CSIC-Universidad de Zaragoza, C/ Pedro Cerbuna 12, 50009 Zaragoza, Spain.

[6]Department of Organic Chemistry, University of Zaragoza, Zaragoza (Spain).

[7]Surgery Department, Medicine Medical School, University of Zaragoza, 50009 Zaragoza, Spain

## EXPERIMENTAL SECTION

**Chemicals**. L-Glutamine (ReagentPlus®, ≥99% (HPLC)), L-Glutamic Acid (ReagentPlus®, ≥99% (HPLC)), L-Aspartic Acid (ReagentPlus®, ≥99% (HPLC)), L-Alanine (ReagentPlus®, ≥99% (HPLC)), L-Proline (ReagentPlus®, ≥99% (HPLC)), L_Glutathione reduced (≥98%), γ-Glu-ε-Lys (≥98.0% (TLC)), Sodium pyruvate (ReagentPlus®, ≥99%), Iron(III) chloride hexahydrate ($FeCl_3{\cdot}6H_2O$, 97%), copper(II) chloride dihydrate ($CuCl_2{\cdot}2H_2O$, 99.0%), sodium acetate anhydrous ($CH_3COONa$, 99.0%), bovine serum albumin (BSA), ethylene glycol (EG), dimercaptosuccinic acid (DMSA, 99.0%), $Na_2HPO_4$, $NaH_2PO_4$, $(CH_3)_3SiCD_2CD_2CO_2Na$ (98% atom D), Chelex resin were purchased from Sigma-Aldrich and were used without further purification. Acetonitrile (HPLC quality) was purchased from VWR chemicals. Dulbecco's modified Eagle's medium (DMEM, Biowest, France) cell culture medium was supplemented with 10% of Fetal Bovine Serum (FBS, GIBCO, USA) and 1% penicillin/streptomycin and 1% amphotericin (Biowest, France)

**Synthesis of the copper-iron oxide nanocatalyst and control nanoparticles.** $CuFe_2O_4$ nanoparticles were prepared following our previous methodologies[1]. The synthesis of both control Cu and Fe oxide control nanoparticles was carried out as follows: 275 mg of BSA were dissolved in 2.5 mL of distilled $H_2O$. Then, 12.5 mL of ethylene glycol were added to the previous mixture. For control copper oxide nanoparticles, 85.5 mg of $CuCl_2{\bullet}2H_2O$ were added to the reaction. In the case of control iron oxide nanoparticles, 275 mg of $FeCl_3{\bullet}6H_2O$ were added to the reaction. Then, the next synthetic steps were the same for both nanomaterials. After adding the metal precursor, 375 mg of sodium acetate were dissolved in the reaction mixture. The reaction was stirred for 2 hours at room temperature. Then, the mixture was transferred to a teflon autoclave and the temperature was increased up to 180 ºC, and maintained overnight. Finally, the solids were purified by centrifugarion (12000 rpm, 20 minutes). To functionalize the nanoparticles with DMSA, 300 mg of DMSA dissolved in 20 mL of $H_2O$

were added to the solid. Then, 5 mL of a 0.5 M NaOH solution were added dropwisely. The final nanoparticle coated with DMSA was purified by centrifugation (12000 rpm, 20 min), resuspended in $H_2O$ and storaged at 4 ºC until further use.

The catalyst was characterized using TEM, HRTEM, STEM-EDS and XRD. TEM images were acquired (FEI TECNAI T20 microscope) operating at 200 keV. Samples were prepared by dropcasting 5 mL of the nanoparticle suspension on a holey carbon TEM grid. High-resolution transmission electron microscopy (HRTEM) was performed using a FEI Titan (80–300 kV) microscope at an acceleration voltage of 300 kV. Sample was prepared by depositing 5 mL of the nanoparticle suspension on a holey carbon TEM grid. X-ray diffraction patterns were obtained in a PANalytical Empyrean equipment in Bragg Brentano configuration using Cu-Kα radiation and equipped with a PIXcel1D detector. TEM images of control CuO nanoparticles and particle size distribution are included in **Figure S16**. TEM images of control iron oxide nanoparticles are included in **Figure S23a**.

**$^1$H-NMR analysis of reaction. General procedure.** Pyruvate, amino acid, Glutathione (GSH) and $CuFe_2O_4$ nanoparticles were added to a sealed vial up to reach a final concentration of 30 mM, 45 mM, 5 mM and 6 mM (expressed in [Cu]), respectively in 10 mL of 1 M $Na_2HPO_4/NaH_2PO_4$. Prior to the addition of nanoparticles, $O_2$ was removed from solution using Ar. Finally, temperature was set up to 37 $^o$C.

For analysis, 1 mL of the sample was collected with a syringe and further filtered using a 0.22 µm Nylon filter. The resulting solution was incubated with 400 mg of Chelex resin for 30 minutes to remove metal ions to avoid paramagnetism in NMR. Then, 50 µL of $D_2O$ containing 20.76 mM of $(CH_3)_3SiCD_2CD_2CO_2Na$ as internal standard, were mixed with 550 µL of the previous solution and were analyzed using a Bruker Avance III 300 spectrometer (Bruker, Billerica, MA, USA) operating at 300 MHz proton frequency. Quantification of produced alanine was carried out using MestRenova software by

integrating -$CH_3$ peak of alanine and normalizing it to 1.76 mM of the internal standard ($(CH_3)_3SiCD_2CD_2CO_2Na$).

**UPLC-MS analysis of reaction. General procedure for all AA except GSH and γ-Glu-ε-Lys.** Pyruvate, amino acid, Glutathione (GSH) and Cu-Fe nanoparticles were added to a sealed vial up to reach a final concentration of 30 mM, 45 mM, 5 mM and 6 mM (expressed in [Cu]), respectively in 2 mL of 1 M $Na_2HPO_4/NaH_2PO_4$. Specifically, for γ-Glu-ε-Lys concentrations employed were x4 times lower (i.e. [γ-Glu-ε-Lys] = 11.25 mM, [Pyruvate] 7.5 mM, [GSH] = 1.25 mM and [Cu] = 1.5 mM) Prior to the addition of nanoparticles, $O_2$ was removed from solution using Ar. Finally, temperature was set up to 37 $^{o}$C.

For analysis, 50 µL of the sample were collected with a syringe and diluted in 50 µL of miliQ $H_2O$. 5 µL of the previous solution were diluted in 995 µL of $H_2O$:Acetonitrile mixture. Resulting solution was filtered and analyzed using a Waters ACQUITY system H-Class coupled to a single quadrupole mass spectrometer with an electrospray ionization (ESI) ACQUITY QDa mass detector. Data acquisition and processing were performed by using MASSLYNX software (Waters Corporation USA). Chromatographic separation was performed using an ACQUITY UPLC BEH Amide column (130 A, 1.7 u, 2.1 mm x 100 mm, Waters). Mobile phase consisted of an initial mixture of Acetonitrile:$H_2O$ (90:10) at a flow rate 0.5 mL·min$^{-1}$, 85 ºC. $H_2O$ composition increased for 3 min until a 65% acetonitrile is reached and then system can recover initial conditions. For analysis of GSH and γ-Glu-ε-Lys reactions, a mobile phase employed was a constant Acetonitrile:$H_2O$ (65:35) at a constant flow rate 0.5 mL·min$^{-1}$, 85ºC.

To determine $k_{obs}$, alanine concentration-time profiles were fitted with Prism9, to a one-phase association equation: $c(t) = (c_{eq})*[1 - \exp(-k_{obs} \cdot t)]$.

On the other hand, initial rate values were also calculated, with $k_{initial}$ corresponding to the slope of a linear fit of the initial production of alanine. The values of $k_{obs}$ and $k_{initial}$ are reported in the graphs of every experiment.

**Intracellular analysis of glutamine, alanine and GSH.** $8 \cdot 10^5$ U251-MG cells were seeded onto P100 dishes. After 24 h, cell media was replaced with DMEM (10% FBS, 1% penicillin/streptomycin and 1% amphotericin) supplemented with $CuFe_2O_4$ nanoparticles (0.05 $mg \cdot mL^{-1}$) for treated cells and left incubated for 24h, 48h or 72h, respectively. Then, cells were washed twice with PBS, trypsinized (5 minutes, 37ºC 5% $CO_2$), centrifuged (300g, 5') and washed again twice with ice-cold PBS (150 rpm , 5'). Finally, cell pellet was resuspended in 300 µL of mili-Q $H_2O$ and ultrasonicated for 30' to ensure a correct cell lysis. Sample for UPLC-MS analysis was prepared by mixing 50 µL of the resulting solution with 950 µL of $H_2O$:ACN mixture (1:1). All samples were filtered with 0.22 mm w/w PFTE filters before injection in UPLC system. Chromatographic separation was performed using an ACQUITY UPLC BEH Amide column (130 A, 1.7 u, 2.1 mm x 100 mm, Waters). Mobile phase consisted of an initial mixture of Acetonitrile:$H_2O$ (90:10) at a flow rate 0.5 $mL \cdot min^{-1}$, 85 ºC. $H_2O$ composition increased for 3 min until a 65% acetonitrile is reached and then system can recover initial conditions.

For GSH, the sample for UPLC-MS analysis was prepared by mixing 30 µL of the resulting solution with 270 µL of $H_2O$ containing 0.1% of formic acid. All samples were filtered with 0.22 µm w/w PFTE filters before injection in UPLC system. Chromatographic separation was performed using an Atlantis Premiere BEH C18AX VG (130 Å, 1.7 µm, 2.1 x 50 mm, WATERS). Column temperature was fixed to 60$^{o}$C. The mobile phase employed consisted in an aqueous solution containing 0.2% of formic acid (v/v) at a flow rate of 0.350 $mL \cdot min^{-1}$.

**Analysis of pyruvate and GSSG in cell media.** $8 \cdot 10^5$ U251-MG cells were seeded onto P100 dishes. After 24 h, cell media was replaced with DMEM (10% FBS, 1% penicillin/streptomycin and 1% amphotericin) supplemented with $CuFe_2O_4$ nanoparticles (0.05 mg·mL$^{-1}$) for treated cells and left incubated for 24h, 48h or 72h, respectively.

For pyruvate analysis, 100 µL of cell media were sampled and mixed with 900 µL of mili-Q $H_2O$, and the resulting mixture was filtered using a 0.22 mm w/w PFTE filter before injection in UPLC system. Chromatographic separation was performed using an ACQUITY UPLC BEH Amide column (130 A, 1.7 u, 2.1 mm x 100 mm, Waters). Mobile phase in channel A consisted of a solution containing 50:50 ACN:$H_2O$ and 10 mM $CH_3COONH_4$ (pH = 9, adjusted with 1 M HCl), while channel B consisted of a solution containing 95:5 ACN:$H_2O$ and 10 mM $CH_3COONH_4$ (pH = 9, adjusted with 1 M HCl). The percentage of channel B during the analysis decreased from 100% to 60% after 0.5 minutes and keep decreasing up to reach a value of 30% after 2 minutes. Then, the percentage of B increased up to 100% until the analysis was finished after 5 minutes. The mobile phase flow rate had a value of 0.4 mL·min$^{-1}$ and the temperature of the column was of 50 ºC.

For GSSG analysis, 100 µL of cell media were sampled and mixed with 900 µL of mili-Q $H_2O$ containing 0.1% of formic acid, and the resulting mixture was filtered using a 0.22 mm w/w PFTE filter before injection in UPLC system. Chromatographic separation was performed using an Atlantis Premiere BEH C18AX VG (130 Å, 1.7 µm, 2.1 x 50 mm, WATERS). Column temperature was fixed to 60ºC. The mobile phase employed consisted in an aqueous solution containing 0.2% of formic acid (v/v) at a flow rate of 0.350 mL·min$^{-1}$.

**Analysis of $CuFe_2O_4$ internalization by confocal microscopy**. Confocal microscopy assay was carried out to assess the capacity of internalization of $CuFe_2O_4$ NPs into U251-MG. Cells were seeded onto 12 mm Ø coverslips, which were deposited on a 24-well plate, at a density of 20 000 cells per well, and incubated at 37 ºC and 5% $CO_2$. After

24 h, cells were treated with $CuFe_2O_4$ NPs dispersed in DMEM at a concentration of 25 μg·mL$^{-1}$ during 24 h (for negative control wells, DMEM was replaced with fresh media). After this time, cells were washed 3 times with DPBS, fixed with 4% paraformaldehyde, and then washed 3 more times with DPBS. In order to prepare the samples for confocal microscopy, cells were permeabilized with 0.1% saponine. After that, samples were deposited onto a drop of Fluoromount-G + DAPI for nuclei staining. Nanoparticle aggregates could be observed due to the reflection of the incident light. To confirm the presence of the nanoparticles inside the cell, a Z-Stack assay of the whole cell, and its ulterior maximum orthogonal projection were performed. This assay was carried out in a confocal microscope (ZEISS LSM 880 Confocal Microscope), using a 63x/1.4 Oil DIC M27 objective.

**Quantification of intracellular copper**. In a 6 well-plate, $2 \cdot 10^5$ cells per well were seeded and incubated at 37ºC under an atmosphere of 5% $CO_2$ for 24 h. Then, the cell culture medium was replaced by a dispersion of $CuFe_2O_4$ nanoparticles in DMEM with a final concentration of 50 μg·mL$^{-1}$. To analyze the internalized copper, cells were detached using a 0.25% Trypsin (X0915-100, Biowest, France) solution in PBS, centrifuged at 6700 g for 5 minutes. The cell pellet was digested with Aqua Regia ($HCl \cdot HNO_3$ 3:1, v/v) overnight and analyzed with an Agilent 4100 MP-AES (Agilent, USA). All samples were filtered using a 0.22 μm nylon filter before their analysis.

**Statistical analysis.** All the results are expressed as mean ± S.E.M. Statistical analysis of the biological experiments and the significant differences among the means were evaluated by t-test using GraphPad Software). Statistically significant differences were express as follows: *p < 0.05, **p < 0.005, ***p < 0.0005 and ****p < 0.00005.

**Computational methods.** The ωB97X-D[46]/6-31+G(d,p)[2] combination was employed to optimize the geometries of stationary points. This functional has a track record of retrieving accurate geometries for systems with non-covalent interactions, such as

hydrogen bonds[3, 4]. To confirm that the optimized geometries were either energy minima or transition states, vibrational frequency calculations were carried out, generating vibrational information to calculate thermochemistry data using *GoodVibes*. Electronic energies were refined using single point energy corrections at the ωB97X-D/Def2-QZVPP[5] level. Furthermore, solvent effects were considered in all calculations using the integral equation formalism variant of the polarizable continuum model (IEF-PCM) with the SMD solvation model (solvent = water)[6].

*Gaussian 16*[7] was used to run all the density functional theory (DFT) calculations. *AQME*[8] was employed to i) generate conformers (program = RDKit)[9], ii) identify and correct errors from geometry optimization and frequency DFT calculations, iii) remove duplicates, and iv) generate input files for single-point corrections in an automated manner (the command lines and input CSV file used are included in the ESI). Molecular representations were created using *PyMOL*[10] with the display settings developed by Dr. Robert S. Paton from Colorado State University, which are openly accessible (https://gist.github.com/bobbypaton).

The calculated vibrational entropies were corrected using quasi-harmonic (QHA) corrections, with a frequency cut-off value of 100.0 $cm^{-1}$, as proposed by Grimme[11]. This correction was performed using the *GoodVibes*[12] program at a temperature of 310.15 K (37 °C). In addition, a correction for the change in standard state from gas phase at 1 atm to a 1 M solution was introduced using the "-c 1" option in *GoodVibes*. The single point energies from ωB97X-D/Def2-QZVPP were corrected using the G corrections computed with ωB97X-D/6-31+G(d,p) to obtain the final G values. This correction was performed using the "--spc SUFFIX" option in *GoodVibes*. All the conformers were considered to calculate the reported Boltzmann-weighted G values of the different reaction steps using the "--pes FILENAME.yaml" option in *GoodVibes*. All these thermochemical values were tabulated in a separate file of the ESI. Automating the

generation of G profiles helped us avoid potential errors resulting from manual manipulation of the data.

## SUPPORTING FIGURES

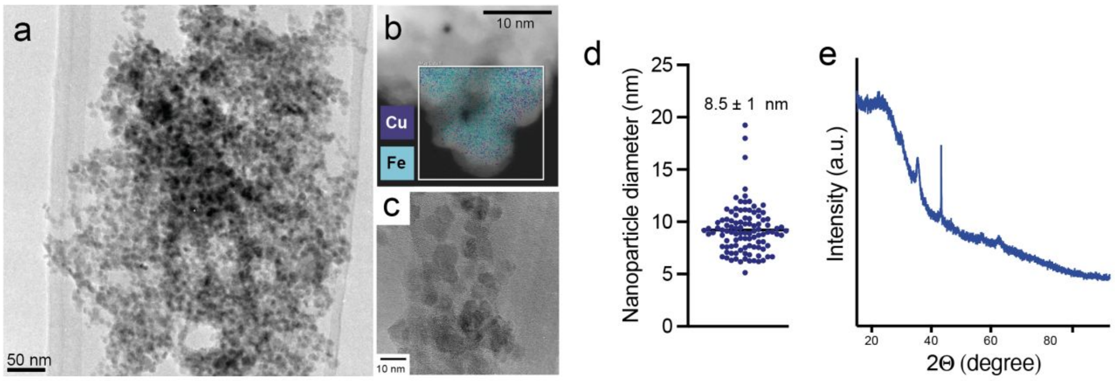


**Figure S1.** Characterization of $CuFe_2O_4$ nanoparticles: (a) TEM images of $CuFe_2O_4$ nanoparticles; (b) HAADF-STEM image and elemental mapping of Cu and Fe; (c) HRTEM image; (d) $CuFe_2O_4$ size distribution (e) XRD pattern of $CuFe_2O_4$ catalyst.

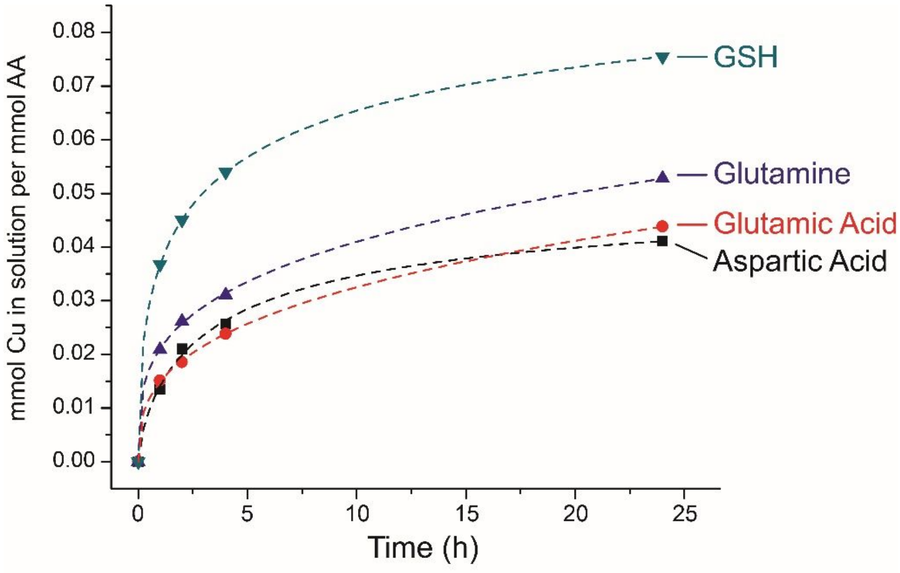


**Figure S2.** Evolution of copper leached from $CuFe_2O_4$ nanoparticles in the presence of different biomolecules evaluated in this work for the transamination reaction including GSH, glutamine, glutamic acid and aspartic acid, normalized per mmol of GSH/AA. Reaction conditions: $[AA]_0$ = 20 mM, $[GSH]_0$ = 5 mM, T = 37 ºC, pH = 7.4 (buffered with TRIS 0.01 M).

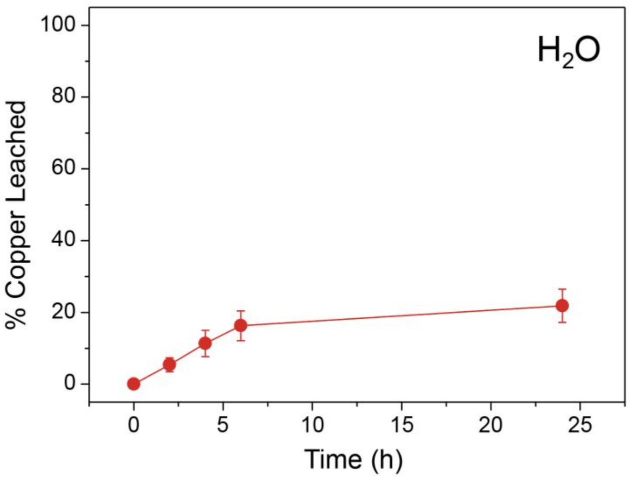


**Figure S3.** Copper release kinetics from $CuFe_2O_4$ nanoparticles in physiological conditions. [$CuFe_2O_4$] = 0.05 mg·mL$^{-1}$, T = 37 ºC, pH = 7.4 (buffered with $NaHCO_3$ 0.01 M).

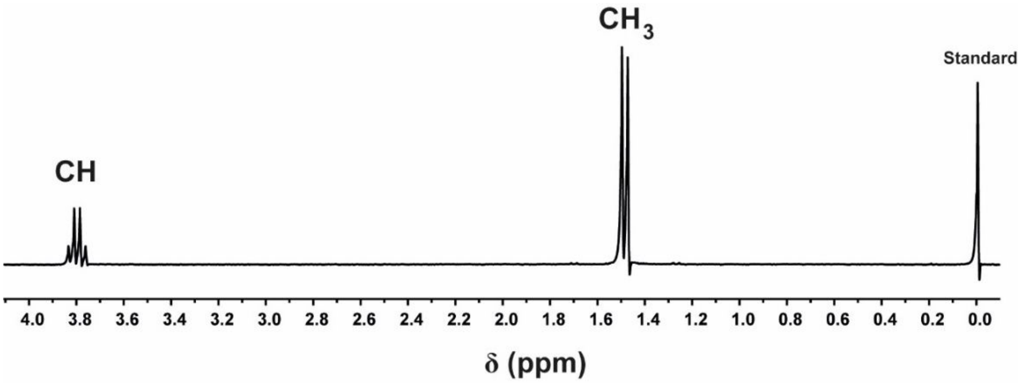


**Figure S4**. $^1$H-NMR spectra of commercial Alanine at pH = 7.4.

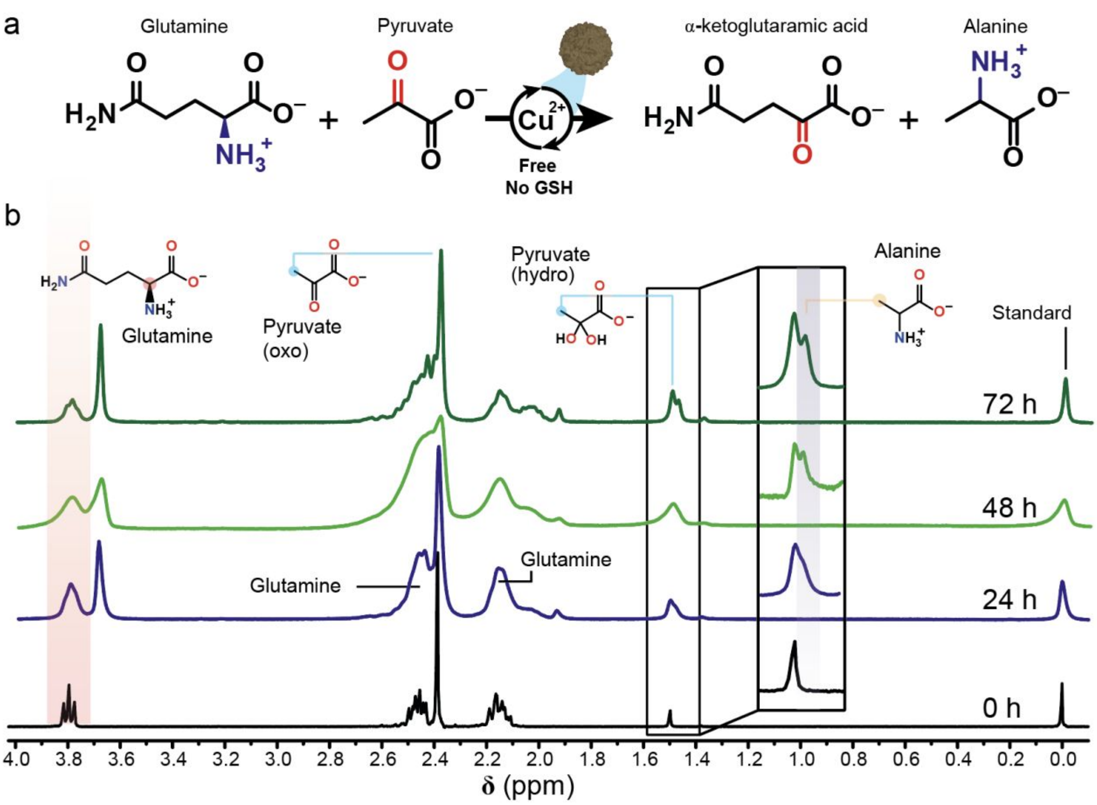


**Figure S5.** (a) Transamination reaction between glutamine and pyruvate catalyzed by $Cu^{2+}$ released from nanoparticles in the absence of GSH. (**b**) $^{1}$H-NMR analysis of glutamine / pyruvate transamination catalyzed by $CuFe_2O_4$ nanoparticles in the absence of GSH reveals the formation of alanine with time. Reaction conditions: [Cu] = 6 mM, [Pyruvate] = 30 mM, [Glutamine] = 45 mM, [GSH] = 0 mM, pH = 7.4 ($Na_2HPO_4$/$NaH_2PO_4$ 1M), T = 37 ºC.

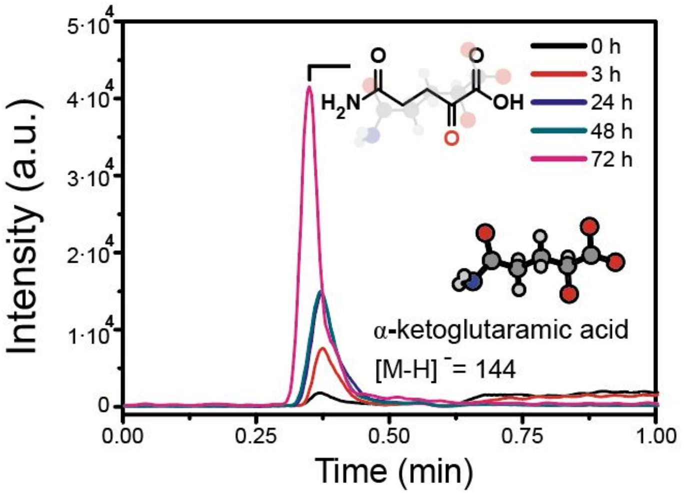


**Figure S6**. UPLC-MS chromatograms of α-ketoglutaramic acid produced in Glutamine/Pyruvate transamination catalyzed by $Cu^{2+}$ released from $CuFe_2O_4$ nanoparticles. Reaction conditions: [Cu] = 6 mM, [Pyruvate] = 30 mM, [Glutamic Acid] = 45 mM, [GSH] = 5 mM, pH = 7.4 ($Na_2HPO_4$/$NaH_2PO_4$ 1M), T = 37 ºC.

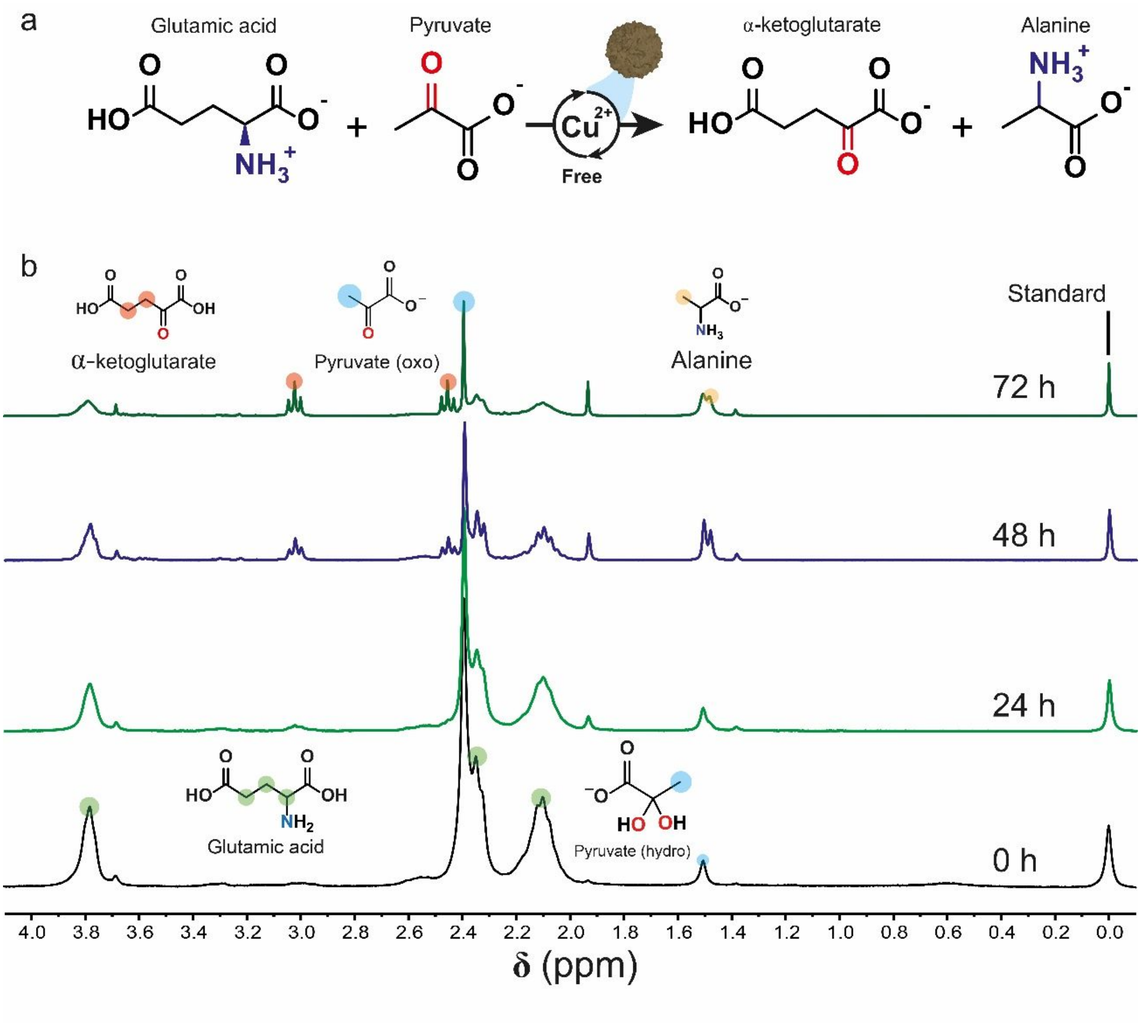


**Figure S7**. (**a**) Transamination reaction between glutamic acid and pyruvate catalyzed by $Cu^{2+}$ released from nanoparticles. (**b**) $^1$H-NMR analysis of glutamic acid / pyruvate transamination catalyzed by $CuFe_2O_4$ nanoparticles reveals the formation of alanine with time. Reaction conditions: [Cu] = 6 mM, [Pyruvate] = 30 mM, [Glutamic Acid] = 45 mM, [GSH] = 5 mM, pH = 7.4 ($Na_2HPO_4$/$NaH_2PO_4$ 1M), T = 37 ºC.

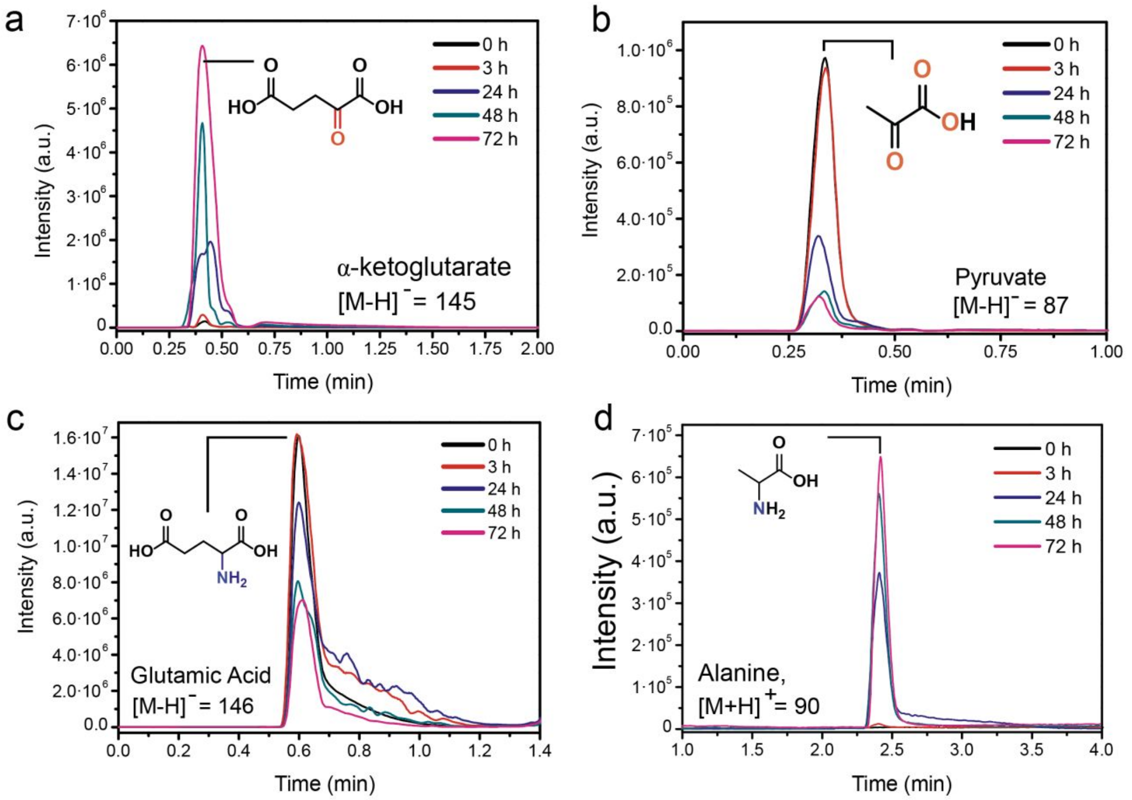


**Figure S8**. UPLC-MS chromatograms analysis of the Glutamic Acid/Pyruvate transamination catalyzed by $Cu^{2+}$ released from $CuFe_2O_4$ nanoparticles UPLC-MS chromatograms of (**a**) α-ketoglutarate, (**b**) pyruvate, (**c**) glutamic acid and (d) alanine show a clear increase in the peak intensity of α-ketoglutarate and alanine and a decrease in the case of pyruvate and glutamic acid. Reaction conditions: [Cu] = 6 mM, [Pyruvate] = 30 mM, [Glutamic Acid] = 45 mM, [GSH] = 5 mM, pH = 7.4 ($Na_2HPO_4$/$NaH_2PO_4$ 1M), T = 37 ºC.

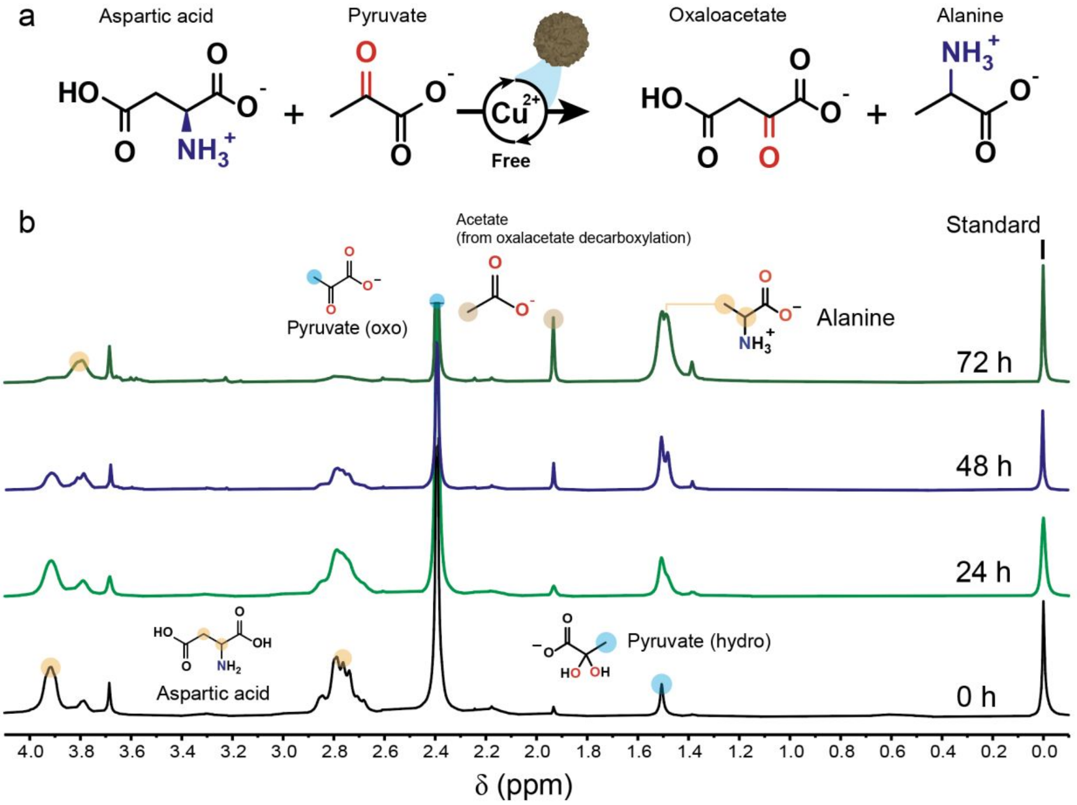


**Figure S9.** (**a**) Transamination reaction between aspartic acid and pyruvate catalyzed by $Cu^{2+}$ released from nanoparticles; (**b**). $^1H$-NMR analysis of aspartic acid / pyruvate transamination catalyzed by CuFe nanoparticles reveals the formation of alanine with time. Reaction conditions: [Cu] = 6 mM, [Pyruvate] = 30 mM, [Aspartic Acid] = 45 mM, [GSH] = 5 mM, pH = 7.4 ($Na_2HPO_4$/$NaH_2PO_4$ 1M), T = 37 ºC.

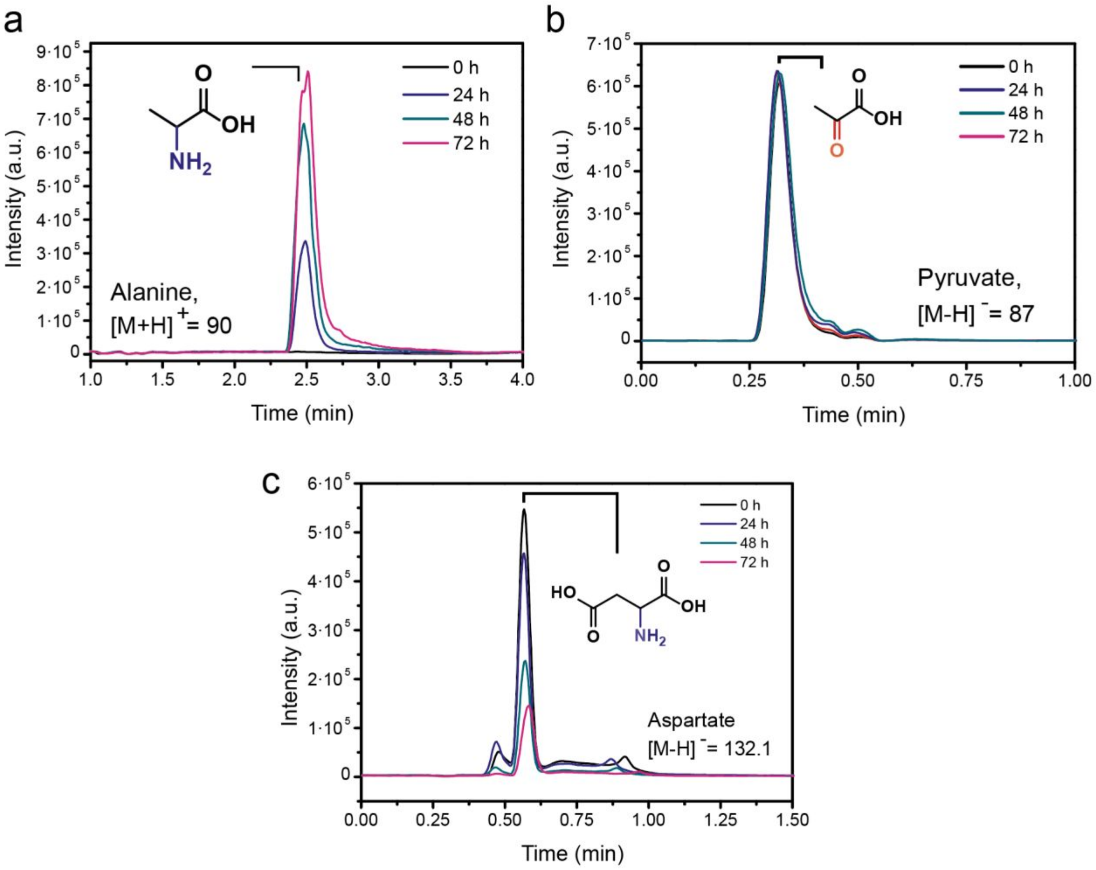


**Figure S10**. UPLC-MS chromatograms analysis of the aspartic acid / pyruvate transamination catalyzed by $Cu^{2+}$ derived from $CuFe_2O_4$ nanoparticles: (a) UPLC-MS chromatograms of Alanine, (b) Pyruvate and (c) Aspartate. Results show an increase in the peak intensity of alanine and a decrease in aspartic acid with time. In this reaction, we were not able to detect the corresponding α-ketoacid likely because it can undergo decarboxylation to pyruvate in the presence of $Cu^{2+}$ [13]. Also, we suggest this may be the reason of not finding a pyruvate decay. Reaction conditions: [Cu] = 6 mM, [Pyruvate] = 30 mM, [Aspartic Acid] = 45 mM, [GSH] = 5 mM, pH = 7.4 ($Na_2HPO_4$/$NaH_2PO_4$ 1M), T = 37 ºC.

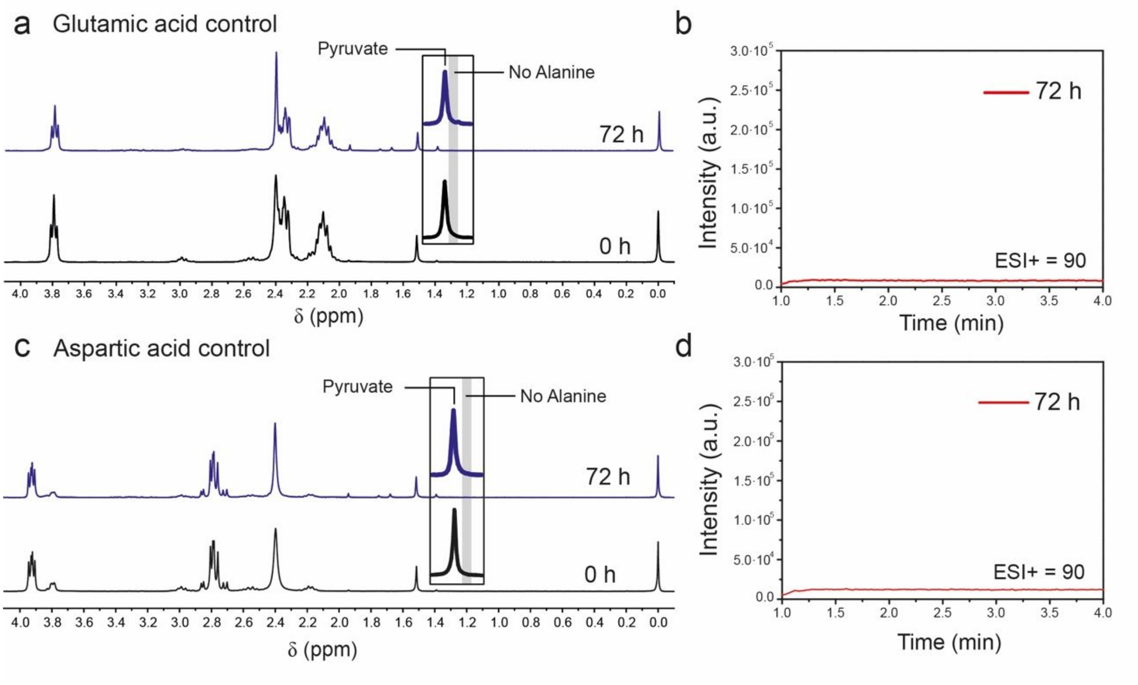


**Figure S11**. Control experiment followed by (**a**) $^1$H-NMR and (**b**) UPLC-MS analysis of the glutamic acid / pyruvate reaction in the absence of the catalyst revealed no alanine formation. (**c**) $^1$H-NMR and (**d**) UPLC-MS analysis of the aspartic acid / pyruvate reaction in the absence of the catalyst revealed no alanine formation. Reaction conditions: [Cu] = 0 mM, [Pyruvate] = 30 mM, [Amino Acid] = 45 mM, [GSH] = 5 mM, pH = 7.4 ($Na_2HPO_4$/$NaH_2PO_4$ 1M), T = 37 ºC.

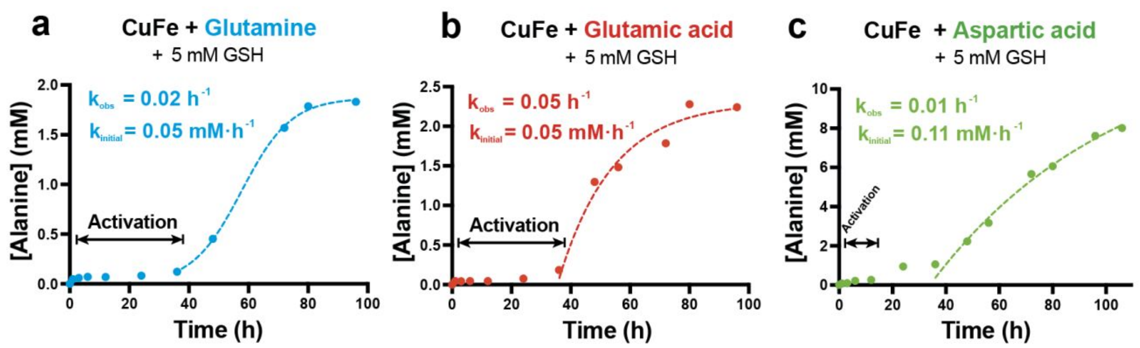


**Figure S12**. $CuFe_2O_4$-assisted production of alanine at different times using (**a**) glutamine, (**b**) glutamic acid and (**c**) aspartic acid quantified by UPLC-MS. Reaction conditions [Cu] = 6 mM, [Pyruvate] = 30 mM, [Amino Acid] = 45 mM, [GSH] = 5 mM, pH = 7.4 ($Na_2HPO_4/NaH_2PO_4$ 1M), T = 37 ºC.

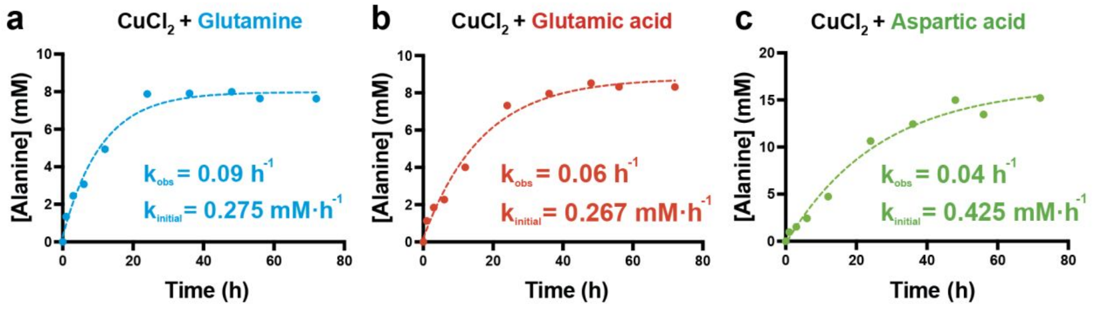


**Figure S13**. Control experiments using $CuCl_2$ as catalyst. Alanine production at different times using (**a**) glutamine, (**b**) glutamic acid and (**c**) aspartic acid quantified by UPLC-MS. Reaction conditions [Cu] = 6 mM, [Pyruvate] = 30 mM, [Amino Acid] = 45 mM, [GSH] = 0 mM, pH = 7.4 ($Na_2HPO_4/NaH_2PO_4$ 1M), T = 37 ºC.

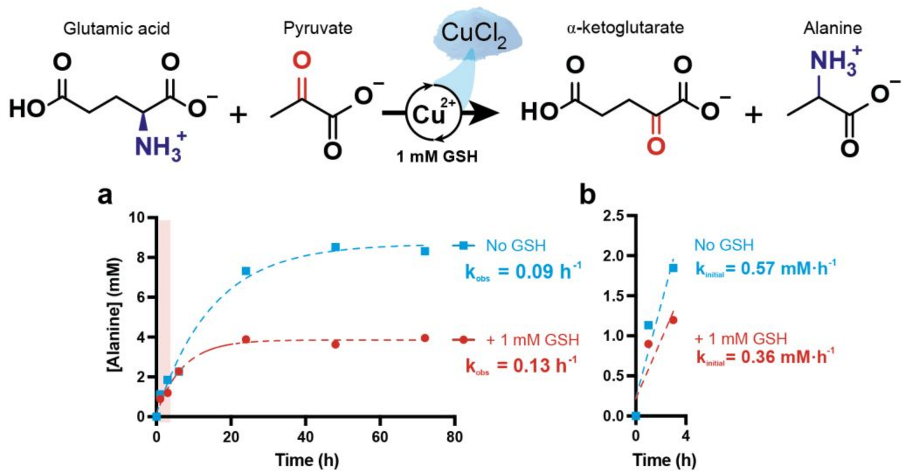


**Figure S14.** Control experiment using $CuCl_2$ as catalyst in the presence of 1 mM GSH using glutamic acid as amino acid substrate. (a) Alanine evolution after 72 h (b) Alanine production at early reaction times. Reaction conditions [Cu] = 6 mM, [Pyruvate] = 30 mM, [Glutamic acid] = 45 mM, $[GSH]_0$ = 1 mM, pH = 7.4 ($Na_2HPO_4/NaH_2PO_4$ 1M), T = 37 ºC.

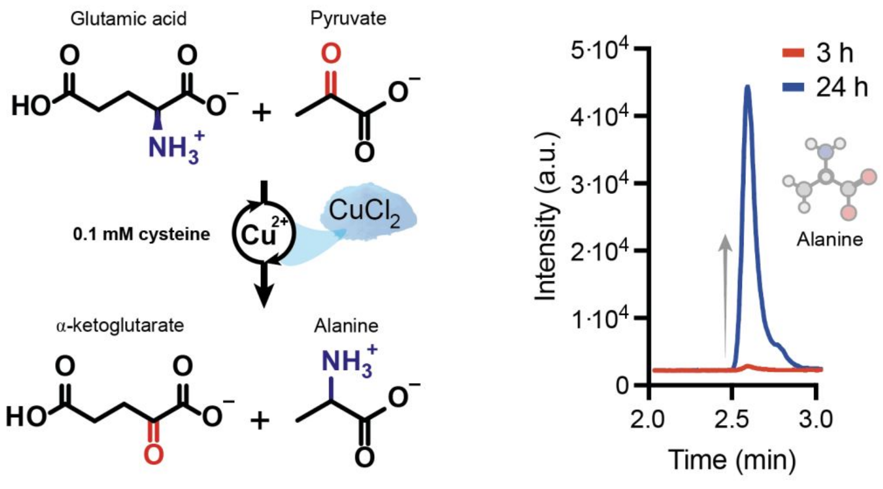


**Figure S15.** Control experiment using $CuCl_2$ as catalyst in the presence of 0.1 mM of cysteine using glutamine as amino acid substrate. Reaction conditions [Cu] = 6 mM, [Pyruvate] = 30 mM, [Glutamine] = 45 mM, $[Cysteine]_0$ = 0.1 mM, pH = 7.4 ($Na_2HPO_4/NaH_2PO_4$ 1M), T = 37 ºC.

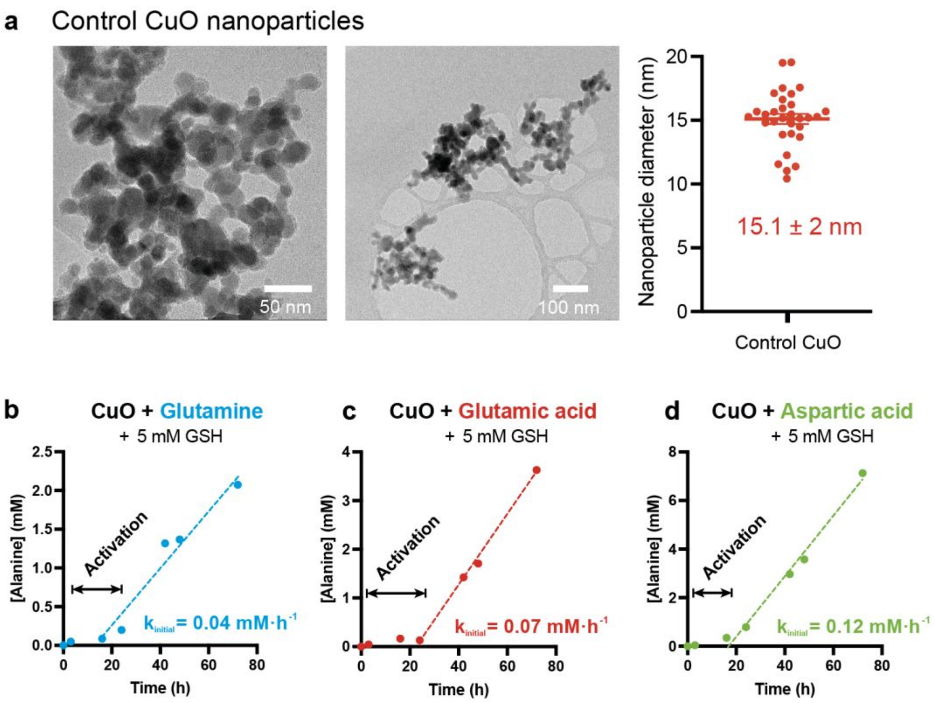


**Figure S16.** (a) Representative TEM images and average diameter of as-prepared control CuO nanoparticles. Production of alanine catalyzed by CuO nanoparticles using (b) glutamine, (c) glutamic acid and (d) aspartic acid as different amino acid substrates. Reaction conditions: [Pyruvate] = 30 mM, [Amino acid] = 45 mM, [GSH] = 5 mM, [Cu] = 6 mM, pH = 7.4 ($K_2HPO_4$/$KH_2PO_4$ buffer 1 M), reaction temperature = 37 ºC.

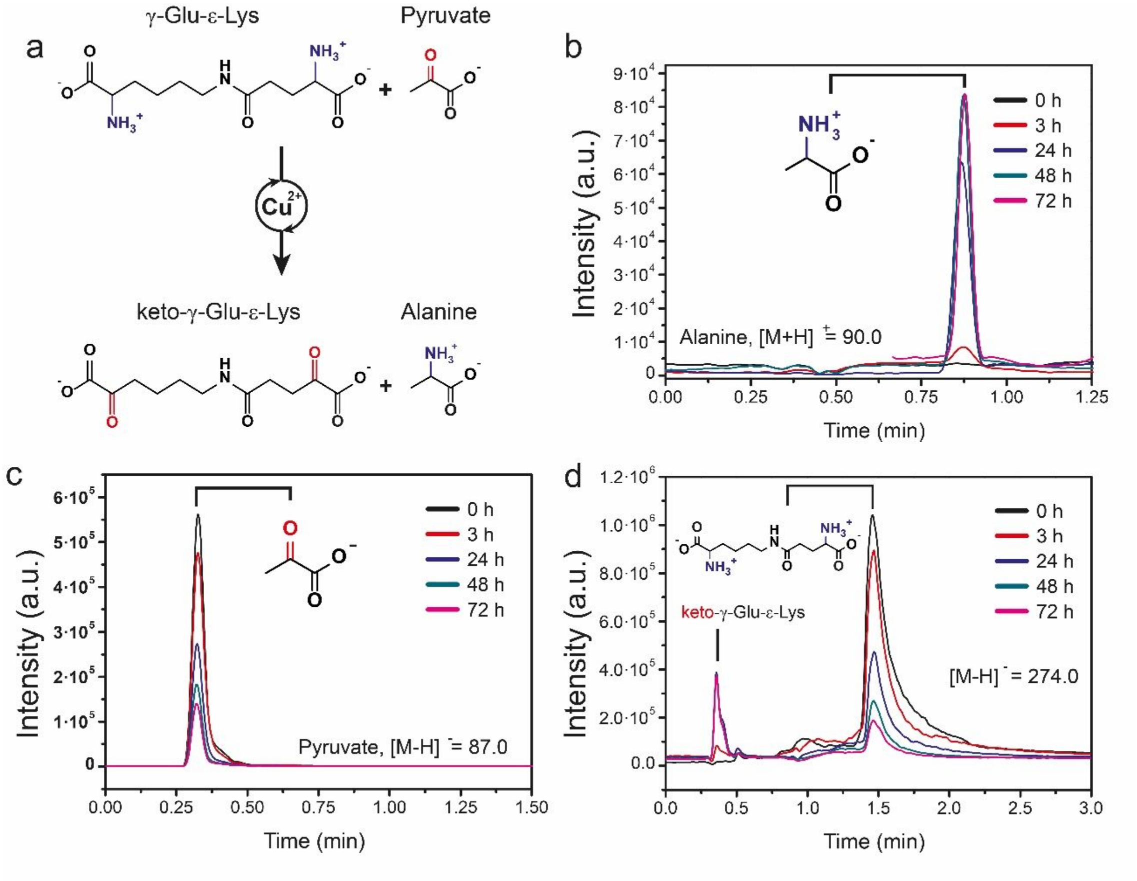


**Figure S17**. (a) Transamination reaction using a dipeptide (i.e. γ-Glu-ε-Lys) as substrate. (b-d) UPLC-MS analysis of the reaction at different times for (b) Alanine, (c) Pyruvate and (d) γ-Glu-ε-Lys revealed the formation of alanine and keto- γ-Glu-ε-Lys whereas pyruvate and γ-Glu-ε-Lys were consumed. Reaction conditions: [Cu] = 2 mM, [Pyruvate] = 7.5 mM, [Amino Acid] = 11.25 mM, [GSH] = 5 mM, pH = 7.4 ($Na_2HPO_4$/$NaH_2PO_4$ 1M), T = 37 ºC. Concentration of amino acid was reduced due to its lower solubility in aqueous solvents.

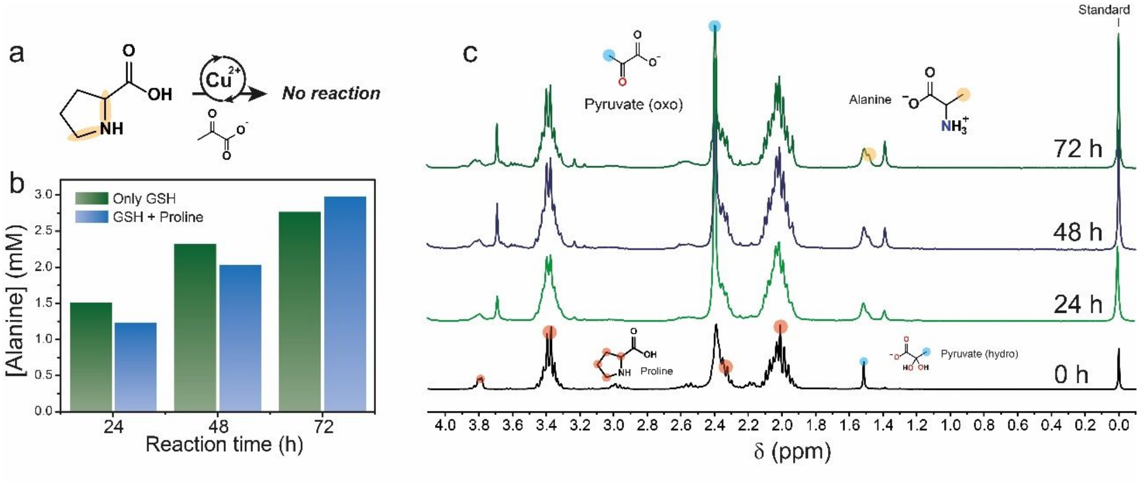


**Figure S18.** Attempt to perform the transamination reaction with pyruvate using proline as amino donor. (a) Proline is the only proteinogenic amino acid that contains a secondary amine. (b) The presence of pyruvate as keto-group source and $Cu^{2+}$ as catalyst did not produce any significant increase in alanine concentration in comparison to control only with GSH. This indicates that only the free $-NH_3^+$ bonded to α-C from glutamic acid residue is a suitable substrate for transamination reaction. (c) $^1$H-NMR spectra of reaction did not show a clear increase in alanine signal ($CH_3$, 1.48 ppm) rather than the generated through GSH-transamination. Reaction conditions [Cu] = 6 mM, [Pyruvate] = 30 mM, [Amino Acid] = 45 mM, [GSH] = 5 mM, pH = 7.4 ($Na_2HPO_4$/$NaH_2PO_4$ 1M), T = 37 ºC.

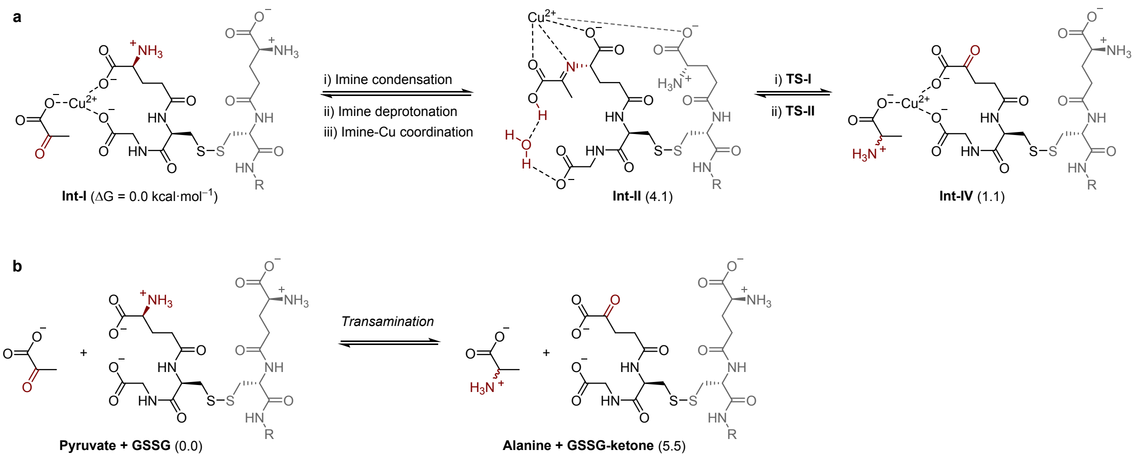


**Figure S19**. Relative Gibbs free energies when modeling GSH and pyruvate (a) in the same calculations and (b) as separated components.

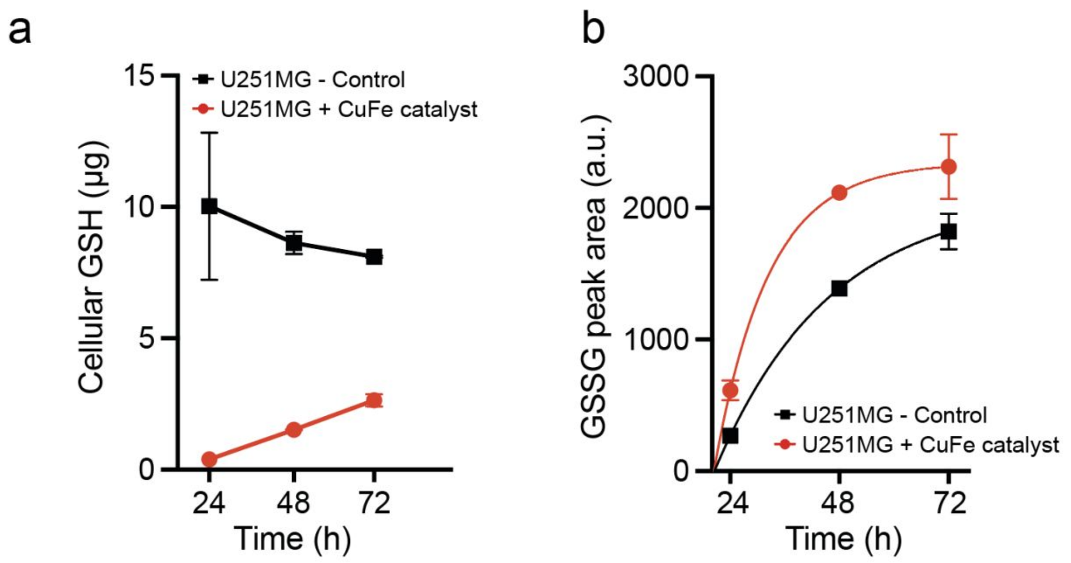


**Figure S20. (a)** Evolution of intracellular GSH after treatment with $CuFe_2O_4$ nanoparticles. **(b)** Variation of GSSG signal in cell media at different incubation times.

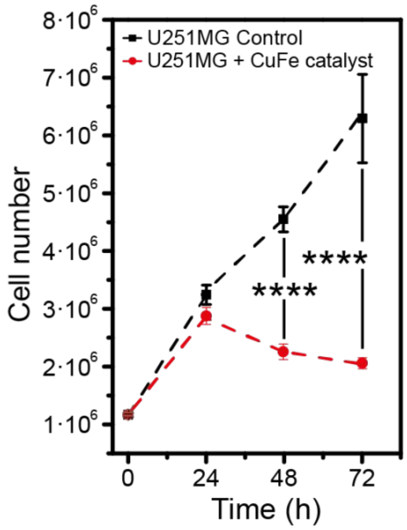


**Figure S21**. Cell number at different incubation times with 0.05 mg·mL$^{-1}$ $CuFe_2O_4$. Cell growth stopped in the presence of $CuFe_2O_4$ nanoparticles, while it increased linearly for the control sample.

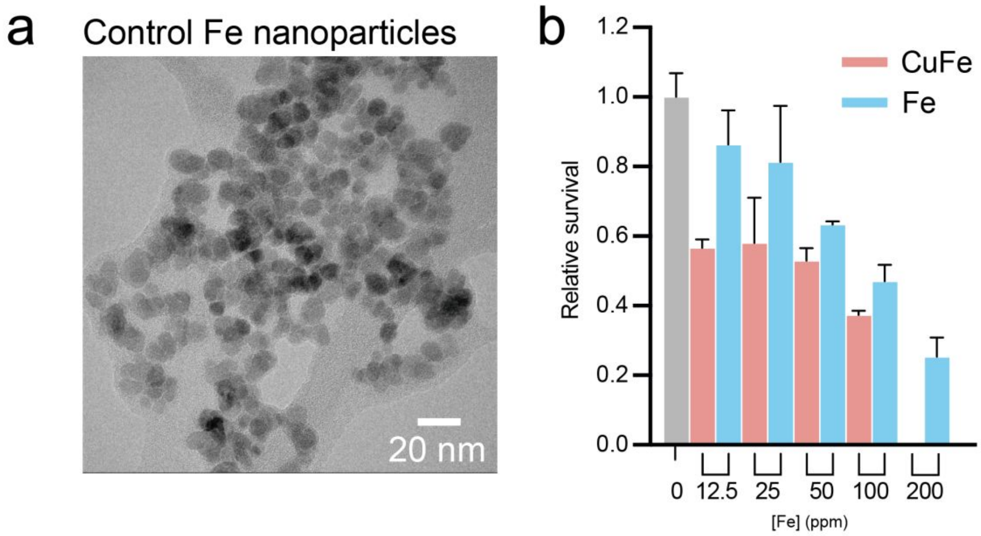


**Figure S22.** (a) TEM image of synthesized control Fe nanoparticles following $CuFe_2O_4$ synthesis protocol but without adding copper precursor (i.e. $CuCl_2·2H_2O$). (b) Comparison of cell viability of U251-MG cell line after the treatment with analogous iron concentration of both $CuFe_2O_4$ and control Fe nanoparticles.

# SUPPORTING DFT INFORMATION

## AQME workflows

*CSEARCH (conformational sampling)*

Input:

The input CSV file (GSSG.csv) contained multiple GSSG···Cu complexes with different types of O···Cu coordination:

| code_name | SMILES |
|---|---|
| **GSSG-Cu_1** | O=C(N[C@H](C(NCC([O-])=O)=O)CSSC[C@@H](C(NC1)=O)NC(CC[C@H]([NH3+])C([O-])=O)=O)CC[C@H]([NH3+])C(O[Cu]OC1=O)=O |
| **GSSG-Cu_2** | O=C(N[C@H](C(NCC([O-])=O)=O)CSSC[C@@H](C(NCC([O-])=O)=O)NC(CC[C@@H]1[NH3+])=O)CC[C@H]([NH3+])C(O[Cu]OC1=O)=O |
| **GSSG-Cu_3** | O=C(N[C@H](C(NCC(O[Cu]O1)=O)=O)CSSC[C@@H](C(NCC([O-])=O)=O)NC(CC[C@H]([NH3+])C([O-])=O)=O)CC[C@H]([NH3+])C1=O |
| **GSSG-Cu_4** | O=C(CC[C@@H](C([O-])=O)[NH3+])N[C@@H](CSSC[C@H](NC(CC[C@@H](C([O-])=O)[NH3+])=O)C(NC1)=O)C(NCC(O[Cu]OC1=O)=O)=O |
| **GSSG-Cu_5** | O=C(N[C@H](C(NCC(O[Cu]1OC2=O)=O)=O)CSSC[C@@H](C(NC2)=O)NC(CC[C@H]([NH3+])C(O1)=O)=O)CC[C@H]([NH3+])C([O-])=O |
| **GSSG-Cu_6** | O=C(N[C@H](C(NCC(O[Cu]1OC2=O)=O)=O)CSSC[C@@H](C(NCC([O-])=O)=O)NC(CC[C@@H]2[NH3+])=O)CC[C@H]([NH3+])C(O1)=O |
| **GSSG-Cu_7** | O=C(N[C@H](C(NCC(O[Cu]12OC3=O)=O)=O)CSSC[C@@H](C(NCC(O2)=O)=O)NC(CC[C@@H]3[NH3+])=O)CC[C@H]([NH3+])C(O1)=O |

Command line:

```
python -m aqme --csearch --input "GSSG.csv" --program rdkit --charge 0 --mult 2 --sample 500
```

*QPREP (generation of input files for initial single-point energy calculations)*

Input:

SDF files from CSEARCH.

Command line:

```
python -m aqme --qprep --files "*.sdf" --qm_input "wb97xd/6-31+g(d,p) scrf=(smd,solvent=water)" --program "gaussian" --mem 16GB --nprocs 8
```

*GoodVibes analysis and creation of reaction steps (sorting initial electronic energies)*

Input files:

Single-point energy calculations executed in Gaussian that come from the previous QPREP step.

The electronic energies of all the conformers from CSEARCH were analyzed with GoodVibes. Then, the most stable conformer was selected, and pyruvate was added manually at different coordination sites to generate the different reaction steps shown in the study. These structures were optimized using DFT with the following Gaussian input:

Intermediates: “opt=calcfc freq=noraman wb97xd/6-31+g(d,p) scrf=(smd,solvent=water) scf=xqc”

Transition states: “opt=(calcfc,ts,noeigen,maxstep=5) freq=noraman wb97xd/6-31+g(d,p) scrf=(smd,solvent=water) scf=xqc”

*QCORR (correction of errors, imaginary frequencies, and duplicates in QM calculations)*

Input files:

OPT+FREQ output calculations from *Gaussian*.

Command line:

```
python -m aqme --qcorr --files "*.log"
```

*QPREP (generation of input files for single-point energy calculations)*

Input:

Successful OPT+FREQ output calculations that passed the QCORR analysis.

Command line:

```
python -m aqme --qprep --files "*.log" --qm_input "wb97xd/def2qzvpp scrf=(smd,solvent=water)" --suffix "QZ" --program "gaussian" --mem 32GB --nprocs 16
```

## Supporting References


1. Bonet-Aleta, J.; Encinas-Gimenez, M.; Urriolabeitia, E.; Martin-Duque, P.; Hueso, J. L.; Santamaria, J., Unveiling the interplay between homogeneous and heterogeneous catalytic mechanisms in copper-iron nanoparticles working under chemically relevant tumour conditions. *Chem Sci* **2022,** *13* (28), 8307-8320.
2. Hehre, W. J.; Ditchfield, R.; Pople, J. A., Self—Consistent Molecular Orbital Methods. XII. Further Extensions of Gaussian—Type Basis Sets for Use in Molecular Orbital Studies of Organic Molecules. *The Journal of Chemical Physics* **2003,** *56* (5), 2257-2261.
3. Chai, J. D.; Head-Gordon, M., Long-range corrected hybrid density functionals with damped atom-atom dispersion corrections. *Phys Chem Chem Phys* **2008,** *10* (44), 6615-20.
4. Goerigk, L.; Grimme, S., A thorough benchmark of density functional methods for general main group thermochemistry, kinetics, and noncovalent interactions. *Physical Chemistry Chemical Physics* **2011,** *13* (14), 6670-6688.
5. Weigend, F.; Ahlrichs, R., Balanced basis sets of split valence, triple zeta valence and quadruple zeta valence quality for H to Rn: Design and assessment of accuracy. *Phys Chem Chem Phys* **2005,** *7* (18), 3297-305.
6. Marenich, A. V.; Cramer, C. J.; Truhlar, D. G., Universal solvation model based on solute electron density and on a continuum model of the solvent defined by the bulk dielectric constant and atomic surface tensions. *J Phys Chem B* **2009,** *113* (18), 6378-96.
7. Frisch, M. J.; Trucks, G. W.; Schlegel, H. B.; Scuseria, G. E.; Robb, M. A.; Cheeseman, J. R.; Scalmani, G.; Barone, V.; Petersson, G. A.; Nakatsuji, H.; Li, X.; Caricato, M.; Marenich, A. V.; Bloino, J.; Janesko, B. G.; Gomperts, R.; Mennucci, B.; Hratchian, H. P.; Ortiz, J. V.; Izmaylov, A. F.; Sonnenberg, J. L.; Williams; Ding, F.; Lipparini, F.; Egidi, F.; Goings, J.; Peng, B.; Petrone, A.; Henderson, T.; Ranasinghe, D.; Zakrzewski, V. G.; Gao, J.; Rega, N.; Zheng, G.; Liang, W.; Hada, M.; Ehara, M.; Toyota, K.; Fukuda, R.; Hasegawa, J.; Ishida, M.; Nakajima, T.; Honda, Y.; Kitao, O.; Nakai, H.; Vreven, T.; Throssell, K.; Montgomery Jr., J. A.; Peralta, J. E.; Ogliaro, F.; Bearpark, M. J.; Heyd, J. J.; Brothers, E. N.; Kudin, K. N.; Staroverov, V. N.; Keith, T. A.; Kobayashi, R.; Normand, J.; Raghavachari, K.; Rendell, A. P.; Burant, J. C.; Iyengar, S. S.; Tomasi, J.; Cossi, M.; Millam, J. M.; Klene, M.; Adamo, C.; Cammi, R.;

Ochterski, J. W.; Martin, R. L.; Morokuma, K.; Farkas, O.; Foresman, J. B.; Fox, D. J. *Gaussian 16 Rev. C.01*, Wallingford, CT, 2016.

8. Alegre-Requena, J. V.; Shree, S. S.; Pérez-Soto, R.; Alturaifi, T. M.; Paton, R. S., AQME: Automated quantum mechanical environments for researchers and educators. *Wires Comput Mol Sci* **2023,** *13* (5), e1663.

9. Landrum, G. J. U. h. w. r. o., https://github. com/rdkit/rdkit, Rdkit: Open-source cheminformatics software. version 2018.03.3.0. **2018,** *149* (150), 650.

10. Schrodinger, L., The PyMOL molecular graphics system. *Version* **2015,** *1*, 8.

11. Grimme, S., Supramolecular Binding Thermodynamics by Dispersion-Corrected Density Functional Theory. *Chemistry – A European Journal* **2012,** *18* (32), 9955-9964.

12. Luchini, G.; Alegre-Requena, J.; Funes-Ardoiz, I.; Paton, R. S. J. F., GoodVibes: automated thermochemistry for heterogeneous computational chemistry data. **2020,** *9*.

13. Mayer, R. J.; Kaur, H.; Rauscher, S. A.; Moran, J., Mechanistic Insight into Metal Ion-Catalyzed Transamination. *Journal of the American Chemical Society* **2021,** *143* (45), 19099-19111.
